# Structure-guided engineering of a polyphosphate kinase 2 class III from an *Erysipelotrichaceae* bacterium to produce base-modified purine nucleotides†

**DOI:** 10.1039/d5cb00108k

**Published:** 2025-07-07

**Authors:** Rachel M. Mitton-Fry, René Rasche, Ann-Marie Lawrence-Dörner, Jannik Eschenbach, Aileen Tekath, Andrea Rentmeister, Daniel Kümmel, Nicolas V. Cornelissen

**Affiliations:** a Institute of Biochemistry, University of Münster, Corrensstr. 36 D-48149 Münster Germany cornelissen@uni-muenster.de; b Department of Chemistry and Biochemistry, Denison University 100 W. College St. Granville Ohio 43023 USA; c Department of Chemistry, Ludwig-Maximilians-University Munich, Butenandtstr. 5-13, Haus F D-81377 Munich Germany

## Abstract

Nucleobase-modified nucleoside-5′-triphosphates (NTPs) are important building blocks for the enzymatic synthesis of non-coding RNAs and mRNAs with improved properties. Chemical phosphorylation of base-modified nucleotides to NTPs remains challenging. Here, we report the enzymatic phosphorylation of purine-modified nucleoside-5′-monophosphates (NMPs) to the corresponding NTPs by the polyphosphate kinase 2 class III from an *Erysipelotrichaceae* bacterium (EbPPK2). The enzyme is highly promiscuous, accepting a range of NMPs with purine modifications. EbPPK2 efficiently catalyses the formation of the corresponding di-, tri- and tetraphosphates, typically with >70% conversion to the NTP. Slower conversion was observed for analogues with oxo- or thio-substitutions at the C6-position. To better understand nucleotide binding and catalysis, we determined the crystal structure of EbPPK2 at 1.7 Å resolution bound to a non-hydrolysable ATP analogue and polyphosphate. This enabled structure-guided design of EbPPK2 variants that efficiently convert GMP analogues, while retaining activity for AMP. Apart from being the preferred industrial-scale ATP recycling catalyst, EbPPK2 and variants bear potential to become the favoured enzyme family for purine-modified NTP production.

## Abstract 2

The polyphosphate kinase 2 class III from an *Erysipelotrichaceae* bacterium is highly promiscuous. We determined the first crystal structure of the enzyme and applied structure-guided engineering to enable efficient access to ATP and GTP analogues.

## Graphical abstract

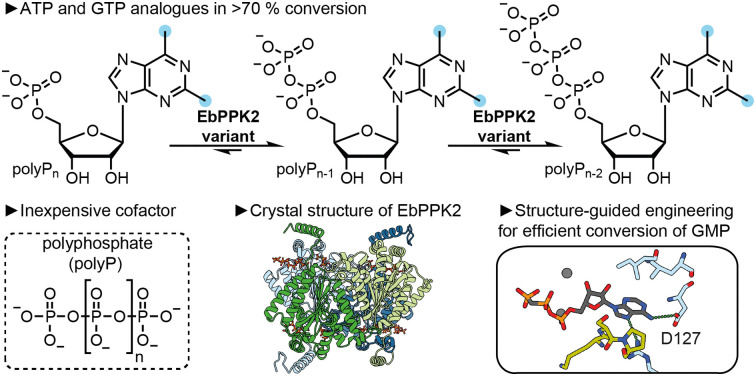

## Introduction

**Fig. 1:**
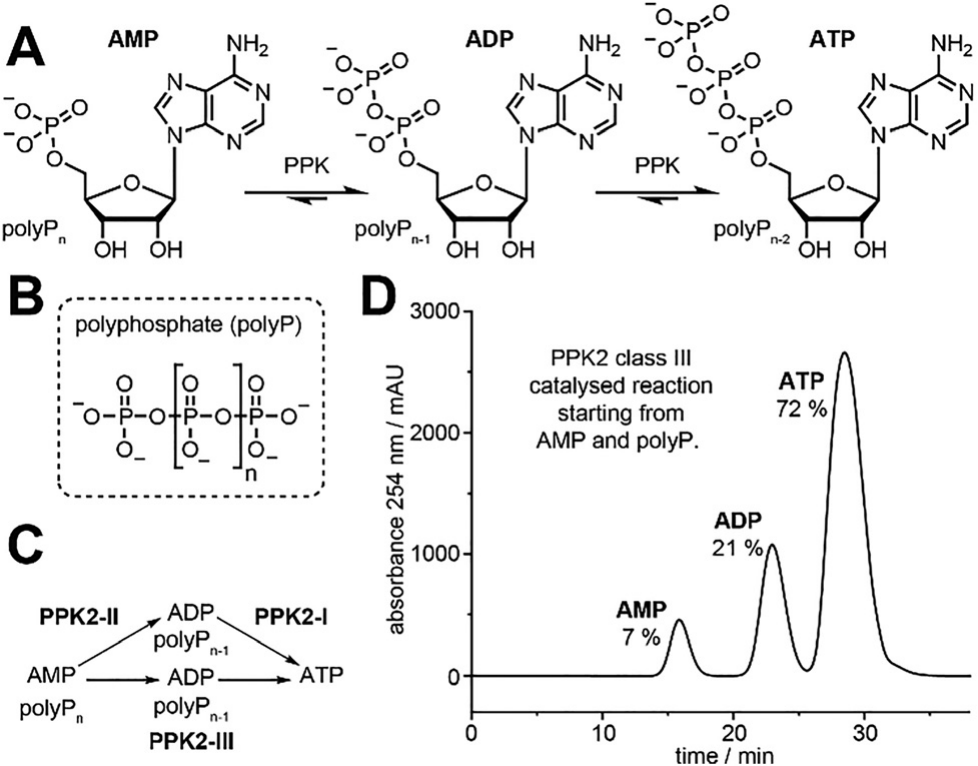
Polyphosphate kinase catalysis. (A) Polyphosphate kinases (PPKs) can use sodium polyphosphate (polyP) to catalyse the reversible phosphorylation of adenosine-5′-monophosphate (AMP) to the corresponding diphosphate (ADP), triphosphate (ATP) and higher phosphorylated species. (B) PolyP is a linear polymer of up to hundreds of phosphate units (*n* = 8 to 800)^9^ found in all domains of life. ^31^P-NMR spectra indicated that the sodium polyphosphate used in this study (Sigma-Aldrich 305553) has an average chain length of 11 phosphate units.^5^ (C) The PPK2 enzymes are subdivided into classes I–III, which preferentially catalyse the reaction from ADP to ATP (class I), the reaction from AMP to ADP (class II) or both reactions (class III). (D) Representative anion-exchange chromatogram of the EbPPK2-catalysed reaction (PPK2 class III). Starting from 5 mM AMP, the reaction reaches the distribution shown in 2 min at 30 °C, with 4 μM EbPPK2 in Tris pH8 and 20 mM MgCl_2_.^5^

Polyphosphate kinases (PPKs) are enzymes that regulate energy metabolism in bacteria and archaea. PPKs catalyse the transfer of phosphate groups between adenosine-5′-triphosphate (ATP), as “energy currency” of the cell, and polyphosphate (polyP), a linear polymer of up to hundreds of phosphate units, as energy storage (Fig. 1A and B). PPKs were initially investigated as targets for antibiotics.^1,2^ These enzymes are also used for biocatalytic ATP recycling,^3^ as well as to produce nucleobase-modified ATP analogues, *e.g.* for mRNA applications.^4,5^ In bacteria, polyphosphate kinases from family 1 primarily catalyse the formation of polyP. These large, membrane associated enzymes are difficult to purify, limiting their biotechnological use.^6^ PPKs from family 2 primarily catalyse production of phosphorylated nucleotides. These PPKs can be subdivided into three classes (I–III) with different substrate preference (Fig. 1C). While PPK2-I enzymes prefer the reaction from ADP to ATP, PPK2-II enzymes favour the reaction from AMP to ADP.^7,8^ The PPK2-III enzymes catalyse both aforementioned phosphorylation reactions, making this subclass the ideal source for enzymes used in ATP recycling or ATP analogue generation (Fig. 1C).

Tavanti *et al.* screened more than 90 PPKs of different classes and identified the class III PPK from an uncharacterised *Erysipelotrichaceae* bacterium (EbPPK2) as an ideal biocatalyst for industrial ATP recycling.^3^ The enzyme was reported to retain good activity after preincubation for 48 h at pH 5–9 or for 4 h in 20% DMSO, conditions that led to inactivation of most of the other PPKs tested.^3^ The reaction catalysed by EbPPK2 is known to approach a distribution of AMP (7%), ADP (21%) and ATP (72%) within 2 min using 4 μM enzyme (Fig. 1D),^5^ reflecting the thermodynamic equilibrium previously described for PPKs.^10^ The enzymatic conversion to the ATP (>70%) often exceeds the yield obtained in chemical phosphorylation (8–30%),^11–14^ while providing a simple, non-hazardous and cost-efficient alternative. Rosenthal and coworkers also used EbPPK2 to convert GMP to GDP and GTP.^15^ Other polyphosphate kinases with a broad substrate scope have been reported^16,17^ or engineered to accept GMP analogues, such as the PPK2 from *Meiothermus ruber* (MrPPK D127S).^18^ Due to its robustness, EbPPK2 is becoming widely used as the preferred ATP recycling system.^3,5,15,19–23^

We recently reported that EbPPK2 has broad specificity and can be used to prepare a wide range of base-modified ATP analogues, including modifications at the N^6^-, C2-, and C8 positions.^5^ Several N^6^- and C2-modified ATP analogues were prepared and purified at the milligram scale for the production of RNAs with hypermodified poly(A) tails.

Here, we aimed to systematically explore the substrate spectrum of EbPPK2 for purine-modified nucleotides. We then determined the first crystal structures of EbPPK2 to better understand nucleotide binding and catalysis. Comparison with crystal structures of other PPK2 class III enzymes revealed high similarities on the structural level, indicating that the high catalytic efficiency and robustness of EbPPK2 cannot be attributed to an obvious feature. These molecular insights additionally enabled us to perform structure-guided design, and the engineered enzymes enabled efficient production of base-modified GTP analogues, while retaining high activity for ATP production.

## Results and discussion

**Fig. 2:**
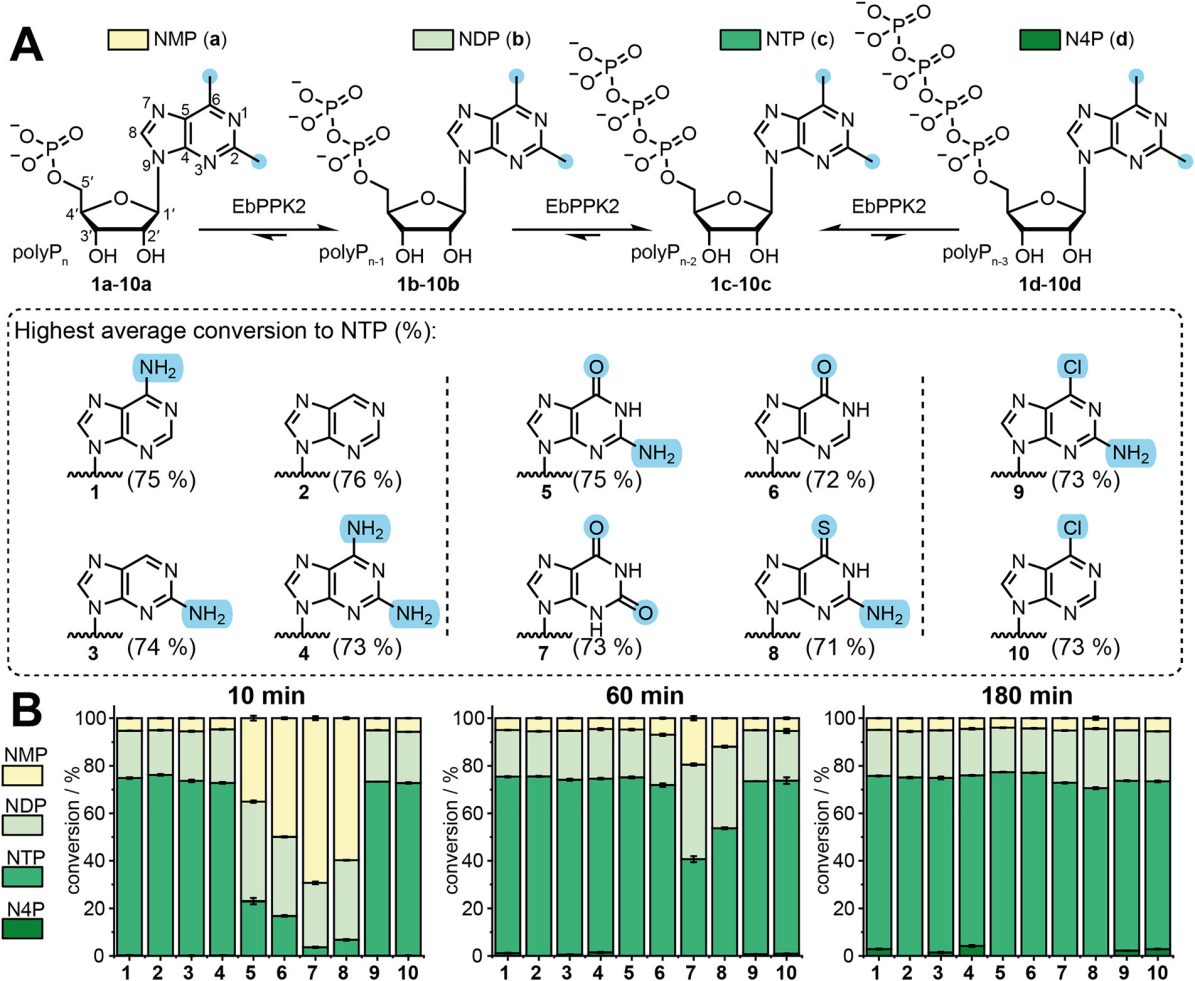
Enzymatic phosphorylation of NMPs by EbPPK2 using sodium polyphosphate. (A) Reaction scheme of the EbPPK2-catalysed reaction. The indicated nucleoside monophosphate (NMP) is converted to the corresponding diphosphate (NDP), triphosphate (NTP) or tetraphosphate (N4P). NMP is labelled according to purine nucleoside numbering. (B) Conversions derived from HPLC analysis after 10 min, 60 min or 180 min as average of three independent experiments with standard deviation (SD). Conditions: 1 mM NMP (1a-10a), 6.1 g L^−1^ sodium polyphosphate, 1 μM EbPPK2 (≙ 0.1 mol%) incubated for 10 min, 60 min or 180 min at 30 °C in a total volume of 40 μL. Buffer: 20 mM Tris pH = 8, 20 mM MgCl_2_.

First, a panel of purine-modified nucleoside-5′-monophosphates (NMPs) was assembled. We chose NMPs that have either amino- or oxo-groups or a hydrogen atom in position C2 or C6 of the purine core, which includes natural AMP and GMP (Fig. 2A). Besides AMP (1a), we used several “AMP-like” analogues (which we define by an amine or hydrogen at the C6-position), such as nebularine monophosphate (2a) and the fluorescent nucleotides 2-aminopurine monophosphate (3a) and 2,6-diaminopurine monophosphate (4a). Besides GMP (5a), we chose several “GMP-like” analogues (defined by the oxo- or thio-substituent at the C6-position): inosine monophosphate (6a), xanthosine monophosphate (7a) and 6-thio-GMP (8a). Additionally, NMPs with chlorine at the C6-position of the nucleobase were investigated. We chose 2-amino-6-chloropurine riboside monophosphate (9a), and we synthesized 6-chloropurine riboside monophosphate (10a) to complete the panel (Fig. S1–S5, ESI†).

We set up enzymatic reactions with 1 mM NMP and 1 μM (0.1 mol%) EbPPK2. The mixture was incubated at 30 °C and pH 8 in the presence of 20 mM Mg^2+^ and 6.1 g L^−1^ polyphosphate (polyP). We previously determined an average chain length of 11 for the polyP by ^31^P-NMR, which corresponds to a concentration of 5 mM for polyP or 55 mM calculated as single phosphates.^5^ Reactions were analysed by either reverse phase or anion exchange HPLC after 10 min, 60 min, and 180 min incubation (Fig. S6–S8 and Table S1, ESI†). To our delight, all of the tested NMPs (1a-10a) were converted to the corresponding NDPs (1b-10b) and NTPs (1c-10c), as summarised in Fig. 2. The identity of the compounds was confirmed by LC-TOF-MS (Fig. S9–S18, ESI†).

Starting from AMP (1a), the reaction approached a distribution of AMP (5%), ADP (20%) and ATP (75%) within 10 min. Incubation for 180 min resulted in minor formation of the corresponding tetraphosphate 1d (3%). To understand how far the reaction can be driven to incorporate additional phosphates, we conducted reactions with higher enzyme concentration and longer reaction times (Fig. S19, ESI†). In this case, EbPPK2 led to the formation of multiple higher phosphorylated nucleotide species, with adenosine-5′-tetraphosphate (A4P) as the biggest fraction. Higher phosphorylated species including A5P, A6P and A7P were found, and adenosine-5′-octaphosphate (A8P) was detectable in trace amounts, as well. This is consistent with a recent report by Matsuura and coworkers that used polyP with an average chain length of 60 and the PPK2-III from *Mangrovibacterium marinum* (MmPPK), detecting polyphosphorylated adenosyl species up to A30P.^17^

The AMP-like analogues 2a-4a were also good substrates for EbPPK2. In 10 min, conversions to the desired NTPs of 76% for 2c, 74% for 3c and 73% for 4c were achieved. GMP and GMP-like analogues were phosphorylated more slowly by EbPPK. The GMP (5a) reaction gave GMP (35%), GDP (42%) and GTP (23%) after 10 min, but reached conversion of GMP (5%), GDP (20%) and GTP (75%) in 60 min. Compounds 6a, 7a and 8a were converted slowly to the desired NTPs 6c (17%), 7c (4%) and 8c (7%) at 10 min. With longer incubation times, conversion >70% were achieved for 6c (72%) at 60 min and for 7c (73%) and 8c (71%) at 180 min. The 6-chloro-containing analogues 9a and 10a were good substrates for EbPPK2, giving 73% conversion to both 9c and 10c in 10 min. A comprehensive table of conversions can be found in the ESI† (Table S1).

Taken together, the “adenine-like” NMPs (1a-4a) and 6-chloro substituted 9a and 10a are very good substrates, reaching 73–75% conversion to the corresponding NTPs in 10 min. In contrast, the “guanine-like” GMP 5a, IMP 6a, XMP 7a, and 6SGMP (8a) are converted much more slowly, reaching only 4% to 23% of NTPs in 10 min, which hampers upscaling of the NTP production to sufficient amounts for the use in mRNA synthesis.

### Crystal structure of EbPPK2

**Fig. 3:**
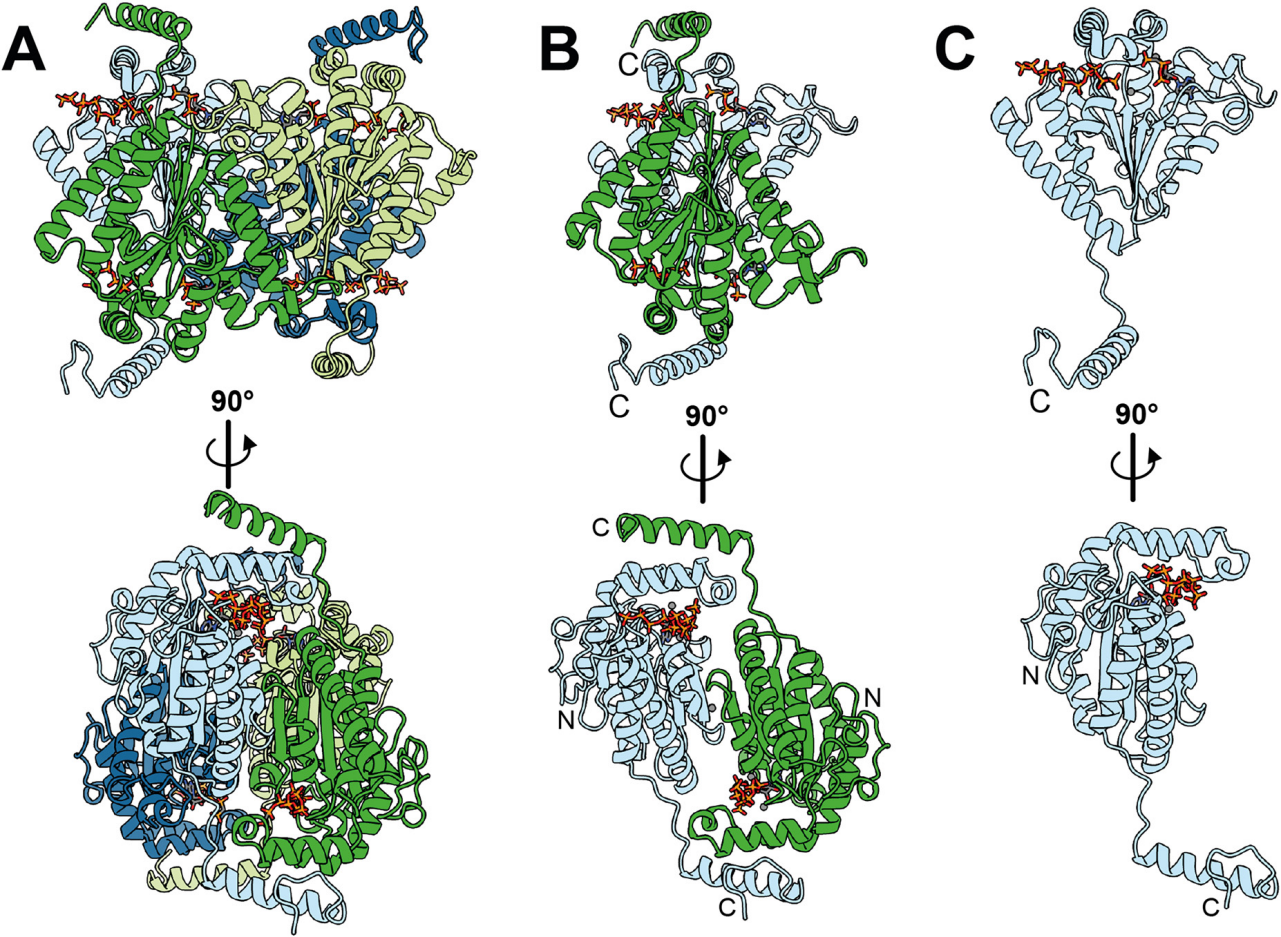
Overall crystal structure of EbPPK2 (PDB: 9IGQ) at 1.7 Å resolution. (A) EbPPK2 forms a homotetramer (dark and light blue, dark and light green). In each of the active sites, adenosine-5′-[(β,γ)-methyleno]triphosphate (ACP) and polyP are bound. (B) The dimer illustrates that a C-terminal α-helix (clamp) connects two monomers. N-terminus and C-terminus are marked with N and C respectively. (C) Each monomer shows the typical V-shape α-β-fold and one active site with a nucleotide-binding cleft and a polyP-binding tunnel in which two Mg^2+^ are bound.

To better understand the observed differences in reactivity between the “adenine-like” *vs.* “guanine-like” bases, we determined two crystal structures of EbPPK2. One structure at 2.3 Å resolution had only polyP bound (PDB: 9IGR), and a second structure at 1.7 Å resolution shows EbPPK2 in complex with the non-hydrolysable ATP analogue adenosine-5′-[(β,γ)-methyleno]triphosphate (ACP) and polyP (PDB: 9IGQ) (Fig. S20 and Table S2, ESI†). Although the structures were determined in different space groups, both are highly similar (root mean squared deviation (RMSD) 0.40 Å, 292 pruned atom pairs, Fig. S20, ESI†). EbPPK2 forms a 146 kDa homotetramer (Fig. 3A), and the EbPPK2 structure closely resembles the structures reported for the PPK2 class III enzymes from *Cytophaga hutchinsonii* (ChPPK2, PDB: 6ANH)^1^ with an root mean square deviation (rmsd) of 0.84 Å over 218 residues and *Meiothermus ruber* (MrPPK, PDB: 5MAQ)^6^ with a rmsd of 0.75 Å over 236 residues.

The catalytic pocket is located on top of the core domain and capped by a lid consisting of three helices. The C-terminus of the core domain is followed by an unstructured linker loop ending in a terminal α-helix that acts as a dimer “clamp” and forms polar interactions with the lid subdomain of another monomer (Fig. 3B). These homodimers are also stabilized by pseudo-β-sheet complementation. The interaction between two dimers is facilitated by a symmetric interaction of a latch-loop and the following αG helix (Fig. S21, ESI†). The core structure is well conserved among other PPK2 class III enzymes, not including the clamp helix and the latch-loop (Fig. S22, ESI†). The overall fold of EbPPK2 monomers resembles the V-shaped P-loop phosphatase fold with a five bladed β-sheet sandwiched between three and four α helices (Fig. 3C).

**Fig. 4:**
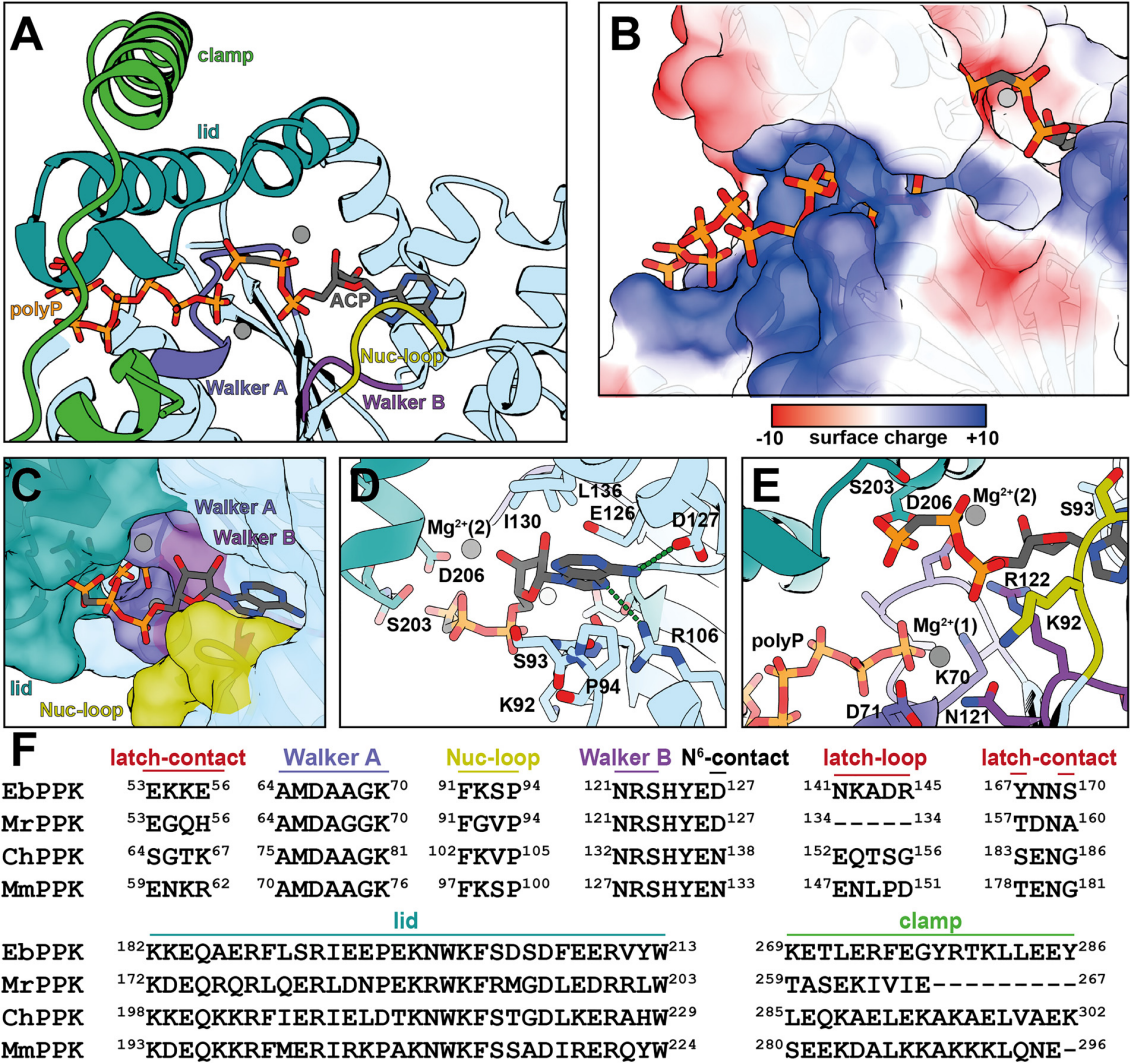
The active site of EbPPK2 (PDB: 9IGQ). (A) The nucleotide-binding pocket of a monomer (light blue) is occupied by ACP (carbon in grey, nitrogen in blue, oxygen in red, phosphorus in orange) and polyP_*n*_ and covered by its lid module (teal). Phosphate-binding motifs Walker A (blue-lilac) and Walker B (lilac) and Nucleotide-binding loop (Nuc-loop, olive) are involved in substrate binding. The clamp helix (green) of the second subunit is located on top of the lid module. (B) PolyP_*n*_ (left) is guided by a basic tunnel towards the active site, which also contains ACP (right). (C) Closeup of the nucleotide bound to the binding pocket confined by the nucleotide loop (Nuc-loop, olive). (D) Coordination of the adenine inside the binding pocket. (E) The active site is flanked by the Walker A and Walker B motifs guiding the incoming polyP_*n*_ and coordinating Mg^2+^ (1), while the ACP 5′-phosphates are held in an inactive conformation by the lid residues and Mg^2+^ (2). (F) Sequence alignment of common motifs found in PPKs for EbPPK2, MrPPK, ChPPK and MmPPK. A complete sequence alignment including DrPPK can be found in Fig. S25 (ESI†).

In both EbPPK2 structures, the lid module covers the active site which includes the Walker A and Walker B motifs (Fig. 4A). These motifs are commonly found in kinases and proteins with nucleotide-binding pockets. The nucleotide-binding cleft, occupied by ACP, positions the 5′-phosphates towards the active site confined by two Mg^2+^ ions (grey). From the opposite direction, a polyphosphate tunnel (Fig. 4A) enables the entrance of polyP into the active site. The basic tunnel, also observed in other PPK2 structures,^1,6^ is defined by positively charged residues, namely K29, K70, R75, R188, R192, K198, K201, R241, K242 and R246 (Fig. 4B). In the EbPPK2/polyP structure, polyP_11_ can be clearly resolved. The terminal phosphate of polyP protrudes into the active site and appears to be optimally positioned for the nucleophilic attack of the terminal phosphate of the nucleotide.

In the ACP-bound structure, the binding pocket is clearly occupied by the nucleoside while the phosphate backbone is less well defined (Fig. S23, ESI†). The active site is enclosed by the lid module and the nucleotide-binding loop (Nuc-loop, Fig. 4C). The nucleobase is bound in a pocket formed by S93/P94 on one and I130/L136 on the other side. R106 contacts the N7 position (3.2 Å) and D127 coordinates the N^6^-position (3.0 Å) of ACP. The C2 position is exposed to solvent (Fig. 4C and D), indicating tolerance for modifications in this position. Notably, the 5′-phosphates are oriented away from the active site and face toward the lid module (Fig. S23, ESI†), not observed in other PPK2 class III structures.^1,6^ Compared to similar PPK2 structures (Fig. S24, ESI†), the lid helix closes the nucleotide pocket tightly, allowing S203 and D206 to interact with the γ-phosphate (Fig. 4E). As a consequence, the ACP γ-phosphate is positioned away from the magnesium ions that catalyse the nucleophilic attack on the terminal phosphate of polyP. We thus hypothesise that the ACP-bound structure represents a catalytically inactive conformation, consistent with the observation that NTPs are poorly phosphorylated by EbPPK2 (Fig. 2).

The Walker A/B motifs as well as the Nuc-loop of EbPPK2 show a high sequence similarity to MrPPK, ChPPK, MmPPK and DrPPK, the PPK from *Deinococcus radiodurans* (Fig. 4F and Fig. S25, ESI†). The N^6^-contact, D127 in EbPPK, is thought to define the substrate tolerance between AMP and GMP, with an aspartate at this position in EbPPK, DrPPK, and MrPPK, which prefer AMP over GMP (Fig. S25, ESI†). Unlike EbPPK, DrPPK and MrPPK appear unable to produce GTP at all.^1,18^ In contrast, enzymes with an asparagine in this position, such as ChPPK and MmPPK, are known to produce GTP.^1,17,18^ Furthermore, mutation of ChPPK N138 to Asp or Glu removed the ability to phosphorylate GDP, while the MrPPK D127S variant gained the ability to produce GTP. Thus, the mere presence of Asp *vs.* Asn at the N^6^-contact position cannot be the sole determinant of substrate specificity.

The latch-loop connects the EbPPK2 homotetramer in the dimer of dimers interface and is a distinct feature that could explain the robustness. In EbPPK2, the latch-loop (N141 to R145) protrudes into the neighbouring monomer with N141 interacting with E53 and K54, A143 interacting with S170, D144 forming a salt bridge with K55 and R145 interacting with Y167 and E56. While a latch-loop is absent in MrPPK and DrPPK, in ChPPK this region is structured and does not tightly interact with the adjacent monomer.

The lid domain of EbPPK2 shows a higher sequence similarity to ChPPK and MmPPK, the PPKs that have a C-terminal clamp helix. Removal of the clamp has been shown to increase the activity with purine nucleotides, while decreasing the activity with pyrimidine nucleotides in ChPPK.^1,17^ Whether this effect can be attributed to lid-clamp interactions, higher flexibility or multimerization remains elusive.

### Enzyme engineering

**Fig. 5:**
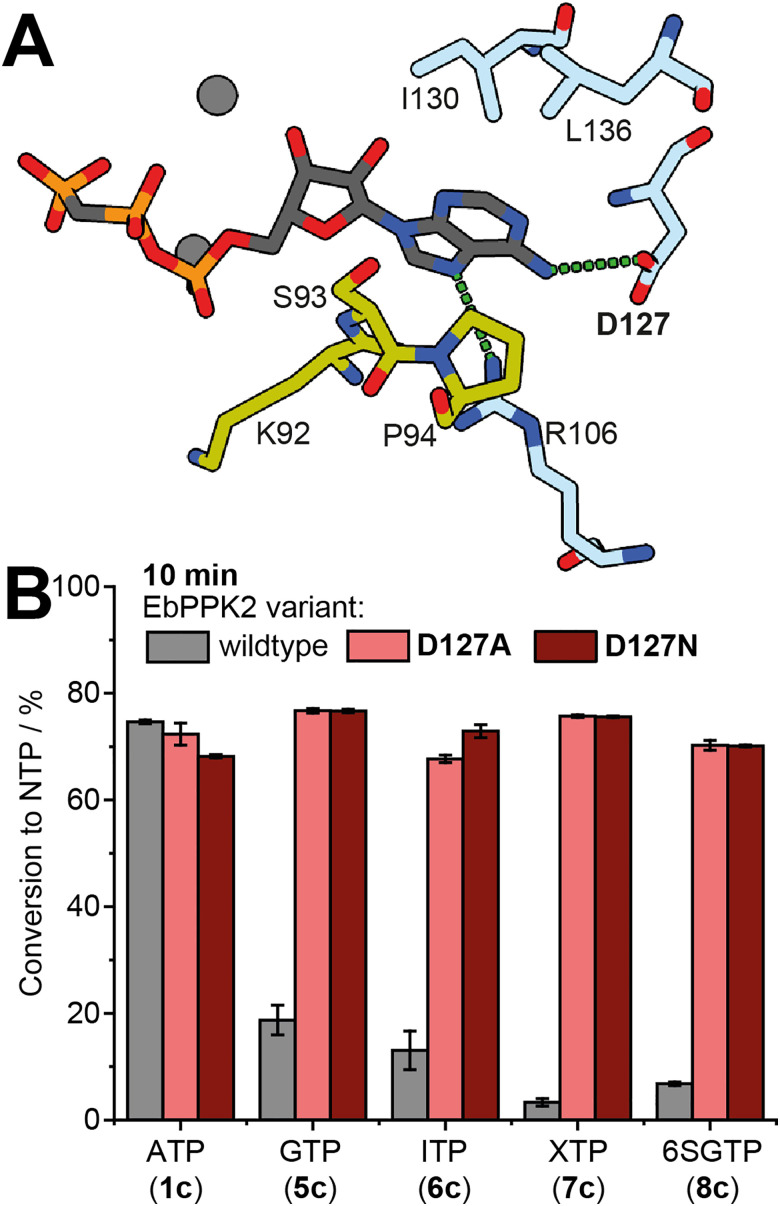
Activity of EbPPK2 variants D127A and D127N. (A) The nucleobase binding pocket of EbPPK2 (PDB: 9IGQ) is shown, highlighting the importance of the interaction between aspartate 127 (D127) and the N^6^-position of adenine. (B) Conversion of wildtype EbPPK2 (grey) is compared to the variants D127A (light red) and D127N (deep red) for ATP (1c), GTP (5c), ITP (6c), XTP (7c) and 6SGTP (8c). Average conversion of three independent experiments is shown with standard deviation (SD). Conditions: 1 mM NMP (1a, 5a-8a), 6.1 g L^−1^ sodium polyphosphate, 1 μM EbPPK2 variant (D127A or D127N) incubated for 10 min or 60 min (Table S3, ESI†) at 30 °C in a total volume of 40 μL. Buffer: 20 mM Tris pH = 8, 20 mM MgCl_2_.

The enzymatic reactions clearly show that purine nucleotide derivatives with O^6^ or S^6^ (“GMP-like” substrates) are less efficiently phosphorylated by EbPPK2. The structural analysis prompted us to generate the EbPPK2 variants D127A and D127N. In D127A, the aspartate in position 127 is replaced with the short amino acid alanine, thereby abolishing electrostatic repulsion, but also removing the opportunity for hydrogen bond formation. In D127N, the newly introduced asparagine can serve as hydrogen bond donor that can stabilize the interactions with O^6^/S^6^-position of the NMP (Fig. 5A).

We expressed and purified the EbPPK2 variants (Fig. S26 and S27, ESI†), confirming identity and purity *via* intact protein LC-TOF-MS (Fig. S28–S30, ESI†). We then tested the variants with AMP and found that the variants retained similar high conversions to ATP (1c) as the 75% observed for the wild-type enzyme (WT-EbPPK2), with 72% for D127A and 68% for D127N in 10 min (Fig. 5B).

Next, we compared WT-EbPPK2 to the variants for the generation of “GTP-like” products GTP (5c), ITP (6c), XTP (7c) and 6SGTP (8c). While WT-EbPPK2 only gave 23% conversion to GTP (5c) in 10 min, the variants showed a faster conversion with 77% for both variants (Fig. 5). This effect was even more pronounced for ITP (6c) production with 17% conversion for WT-EbPPK2 and 68% and 73% for D127A and D127N, respectively. The greatest increase was observed for XTP (7c), with 4% conversion with WT-EbPPK2 and 76% for both variants. Both variants also led to faster production of 6SGTP (8c) with WT-EbPPK2 giving 7% conversion and D127A and D127N both giving 70% in 10 min. Representative HPLC chromatograms and conversions are found in the ESI† (Fig. S31, S32 and Table S3).

These results also confirm that hydrogen bonding capability is not essential for stabilizing the interaction between the 6-position of the substrate and the enzyme, as D127A was seen to have similar behaviour with AMP, as well as all four GMP-like substrates. This supports results seen with MrPPK, where the corresponding D127A and D127S mutations were observed to afford equivalent conversion of GMP to GTP.^18^ Interestingly, the MrPPK D127N mutation showed slightly lower production of GTP, despite offering the potential to serve as a hydrogen bond donor. The MrPPK D127S mutant was further tested against IMP, XMP and 6SGMP, with MrPPK D127S producing ∼45–70% conversion at 0.5 mM substrate concentration with 4 μM enzyme in 20 min at 50 °C.^18^ In comparison, EbPPK2 D127N gives >70% conversion to the NTPs starting from 1 mM substrate with 1 μM enzyme in 10 min at 30 °C.

A highly efficient EbPPK variant will enable preparative reactions in the future, as well as the exploration of the chemical space of GTP analogues for novel ligands for the study of G-proteins^24^ or GTP analogues for the modification of the mammalian mRNA 5′-cap.^25^

## Conclusions

EbPPK2 is a class III polyphosphate kinase that accepts a surprisingly broad range of substrates. We systematically studied the effect of substituents at the C6-position of purine-modified NMPs for EbPPK2 catalysis and found that amino-, chloro- groups and hydrogen atoms are strongly favoured over thio- and oxo- substituents. A weaker effect was observed for the C2-position with amino > H > oxo.

To better understand nucleobase recognition, we solved the first crystal structures of EbPPK2, both as a complex with polyP (2.3 Å) and as a ternary complex with polyP and the non-hydrolysable ATP analogue ACP (1.7 Å). The enzyme forms a homotetramer with four active sites and a polybasic patch which guides the polyP through a tunnel to the active site adjacent to the Walker A motif. Consistent with its broad specificity, the nucleobase-binding cleft is rather unspecific, with a solvent-exposed C2-position, N7 of the adenine ring interacting with R106 and the N^6^-position coordinated by D127.

We thus created EbPPK2 variants D127A and D127N, which retain the ability to phosphorylate AMP, while gaining high activity for production of base-modified GTP analogues, a substance class where chemical synthesis is troublesome.^13,26,27^

Overall, this work provides fundamental insights into polyphosphate kinase catalysis and enables the efficient, simple and sustainable production of various purine-modified NTPs for the application in non-coding RNA or mRNA in the future.

## Author contributions

R. M. M.-F. and N. V. C. conceived the project. R. M. M.-F., R. R., A.-M. L. D., J. E., A. T. and N. V. C. designed and performed experiments. R. R. and D. K. performed protein crystallization and structure analysis. All authors discussed the results. A. R. and D. K. secured funding. R. M. M.-F. and N. V. C. wrote the manuscript. All authors discussed and agreed on the final version.

## Conflicts of interest

The authors declare no competing interests.

## Supplementary Material

CB-006-D5CB00108K-s001

## Data Availability

The data supporting this article have been included as part of the ESI.†
