# Supplementary material for: Structure-guided engineering of a polyphosphate kinase 2 class III from an Erysipelotrichaceae bacterium to produce base-modified purine nucleotides
Source: RSC Chem Biol. 2025 Jul 7;6(8):1328–35. doi: 10.1039/d5cb00108k (PMC12257356; doi:10.1039/d5cb00108k)
Supplement: CB-006-D5CB00108K-s001 [file CB-006-D5CB00108K-s001.pdf]

Supplementary Information (SI) for RSC Chemical Biology.
This journal is © The Royal Society of Chemistry 2025

# Supporting Information

## Structure-Guided Engineering of a Polyphosphate Kinase 2 Class III from an *Erysipelotrichaceae* Bacterium to Produce Base-Modified Purine Nucleotides

Rachel M. Mitton-Fry[a,b], René Rasche[a], Ann-Marie Lawrence-Dörner[a], Jannik Eschenbach[a], Aileen Tekath[a], Andrea Rentmeister[c], Daniel Kümmel[a], Nicolas V. Cornelissen[a,*]

[a]Institute of Biochemistry, University of Münster, Corrensstr. 36, D-48149 Münster, Germany.

[b]Department of Chemistry and Biochemistry, Denison University, 100 W. College St., Granville, Ohio 43023, USA

[c]Department of Chemistry, Ludwig-Maximilians-University Munich, Butenandtstr. 5-13, Haus F, D-81377 Munich, Germany

*E-mail: cornelissen@uni-muenster.de

# Table of contents

Materials and methods ........ 3

HRMS ........ 3

NMR ........ 3

HPLC anion exchange method A ........ 3

HPLC reverse phase method B ........ 4

LC-TOF-MS of nucleotides ........ 4

LC-TOF-MS of intact proteins ........ 5

Synthesis of 6-chloropurine riboside 5′-monophosphate (**10a**) ........ 6

Polyphosphate kinase 2 from *Erysipelotrichaceae* bacterium (EbPPK2) ........ 7

Site-directed mutagenesis ........ 8

EbPPK2 reactions ........ 11

Protein crystallization ........ 11

Supplementary figures and tables ........ 12

Synthesis of 6-chloropurine riboside 5′-monophosphate (**10a**) ........ 12

HPLC analysis of EbPPK2-catalysed reactions ........ 15

Table S1: Conversions of EbPPK2 reactions starting from **1a-10a**. ........ 18

LC-TOF-MS analysis of EbPPK2 catalysed reactions ........ 19

Crystal structure of EbPPK2 ........ 31

Table S2: Data collection and refinement statistics. ........ 31

Expression and purification of EbPPK2 variants D126A and D127N ........ 36

HPLC analysis of EbPPK2 variant catalysed reactions ........ 40

Table S3: Conversions of EbPPK2 variants D127A or D127N. ........ 42

## Materials and methods

All chemicals and reagents were purchased from Sigma-Aldrich, Acros Organics, VWR, TCI Chemicals, BLD Pharmatech, BIOLOG Life Science Institute and Jena Bioscience and were used without further purification unless otherwise stated. Graham´s salt (Sigma-Aldrich 305553) was used as the polyphosphate source.

### HRMS

HRMS were measured on an Orbitrap Exploris™ 120 mass spectrometer. Samples were dissolved in MeOH and ionized by electrospray ionization.

### NMR

NMR spectra were measured at 299 K on a Bruker Neo 400 spectrometer. The chemical shifts (δ) were reported in ppm relative to deuterated solvents as internal standard ($D_2O$ = 4.79 ppm).

### HPLC anion-exchange method A

| | |
|---|---|
| Buffer A | dd$H_2O$ |
| Buffer B | 200 mM $NaH_2PO_4$, pH = 2.0 |
| Buffer C | Acetonitrile |
| Flowrate | 0.8 mL/min |
| Temperature | 20 °C (column oven) |
| column | Amaze HA 3x50 mm, 3 µm, 100 Å<br>Helix Chromatography AHA-300531 |

| Min | %A | %B | %C |
|---|---|---|---|
| 0 | 50 | 30 | 20 |
| 0.5 | 50 | 30 | 20 |
| 2 | 0 | 80 | 20 |
| 4 | 0 | 80 | 20 |
| 4.5 | 50 | 30 | 20 |
| 7 | 50 | 30 | 20 |

Note that with anion-exchange separation, the less anionic analyte elutes first, and the general order of elution is NMP, NDP, NTP and N4P.

## HPLC reverse phase method B

| | | | Min | %A | %B |
|---|---|---|---|---|---|
| Buffer A | 50 mM $NH_4OAc$, pH = 6.0 | | 0 | 100 | 0 |
| Buffer B | Acetonitrile | | 1 | 100 | 0 |
| Flowrate | 1 mL/min | | 5 | 0 | 100 |
| Temperature | 20 °C (column oven) | | 6.5 | 0 | 100 |
| column | Poroshell 120 EC-C18 column | | 7 | 100 | 0 |
| | 3 x 50 mm (2.7 µm) | | 10 | 100 | 0 |
| | Agilent P.N. 699975-302 | | | | |

Note that with reverse-phase separation, the most polar analyte elutes first, and the general order of elution is N4P, NTP, NDP and NMP.

## LC-TOF-MS of nucleotides

LC-TOF-MS were performed on Bruker maXis II ultra-high resolution QTOF coupled to a Thermo Scientific UltiMate 3000® UHPLC.

| | | | Min | %A | %B |
|---|---|---|---|---|---|
| Buffer A | 50 mM $NH_4OAc$, pH = 6.0 | | 0 | 100 | 0 |
| Buffer B | Acetonitrile | | 1 | 100 | 0 |
| Flowrate | 1 mL/min | | 5 | 0 | 100 |
| Temperature | 20 °C (column oven) | | 6.5 | 0 | 100 |
| column | Poroshell 120 EC-C18 column | | 7 | 100 | 0 |
| | 3 x 50 mm (2.7 µm) | | 10 | 100 | 0 |
| | Agilent P.N. 699975-302 | | | | |

Note that with reverse-phase separation, the most polar analyte elutes first, and the general order of elution is N4P, NTP, NDP and NMP.

**LC-TOF-MS of intact proteins**

Mass analysis of intact proteins was performed using an UltiMate™ 3000 RS system (Thermo Fisher Scientific GmbH, Dreieich, Germany) connected to a maXis II UHR-qTOF mass spectrometer (Bruker Daltonik GmbH, Bremen, Germany) with a standard ESI source (Apollo, Bruker Daltonik GmbH, Bremen, Germany). Proteins were reduced with 2 mM TCEP for 15 min on ice. Samples were acidified using a 5 % formic acid solution to achieve pH 2-3 and centrifuged (14000 rpm, 2 min). According to the protein concentration, an appropriate volume of the supernatant was loaded on a C4 column (Advance Bio RP-mAb C4, 2.1 mm x 50 mm, 3.5 µm, Agilent Technologies, Waldbronn, Germany) at a flow rate of 0.3 mL/min in 5 % eluent B (eluent A: 0.1 % formic acid in water; eluent B: 0.1 % formic acid in acetonitrile). After a desalting period of 3 minutes at 5 % B, a steep gradient was applied (5-100 % B in 3 min), followed by 1 min at 100 % B, 1 min at 100 % B and 2 min at 5 % B. MS settings: capillary voltage 4500 V, end-plate offset 500 V, nebulizer 5.0 bar, dry gas 9.0 L/min, dry T = 200 °C, mass range m/z 300-3000. Data were analyzed with DataAnalysis 4.4 (Bruker Daltonik GmbH, Bremen, Germany) and deconvolution was performed using the MaxEnt algorithm implemented in the software.

## Synthesis of 6-chloropurine riboside 5′-monophosphate (10a)

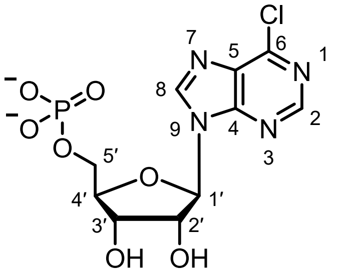


6-chloropurine riboside (57 mg, 0.2 mmol, 1 eq.) was dissolved in 20 mL dry trimethyl phosphate (TMP) under an argon atmosphere. The solution was cooled to 0 °C and freshly distilled $POCl_3$ (1.6 eq.) was added dropwise. After stirring for 3 hours, the reaction was quenched with an aqueous solution of triethylammonium bicarbonate (TEAB, 1 M, 35 mL) until the gassing stopped and stirred for another 10 min. To remove residual TMP, the solution was washed with methyl *tert*-butyl ether (MTBE, 5 x 60 mL), ethyl acetate (1 x 60 mL) and MTBE (5 x 60 mL). Afterwards, residual ether was removed from the aqueous phase through argon bubbling for 15 min. Subsequently, the solution was concentrated under vacuum to a total volume of approximate 10 mL. The purification of was performed by C18 flash chromatography on puriFlash XS520 (Interchim), with a C18-AQ HP Gold 100 g column (Teledyne ISCO).

Conditions of the preparative purification of **10a**.

| | |
|---|---|
| Buffer A | 50 mM $NH_4OAc$, pH = 6 |
| Buffer B | Acetonitrile |
| Flowrate | 60 mL/min |
| Temperature | room temperature |
| column | C18-AQ HP Gold 100 g |

| Min | %A | %B |
|---|---|---|
| 0 | 100 | 0 |
| 4 | 100 | 0 |
| 16 | 50 | 50 |
| 17 | 0 | 100 |
| 20 | 0 | 100 |

The compound was further purified via ion-exchange chromatography. 10 g of Dowex 50WX8 ion-exchange resin (Thermo Scientific) was washed with 40 mL $ddH_2O$ and activated with 40 mL of NaOH (2 M) by stirring for 20 minutes. The suspension was poured into a 12 mL syringe containing a cotton plug. The crude product solution was added on top of the resin and rinsed through it with $ddH_2O$. Fractions containing the desired product were identified by spotting on a thin-layer chromatography plate for visualization under a UV-lamp. These fractions were pooled and lyophilized. The product was obtained as a white solid (50 mg, 0.13 mmol, 65 %).

**FTMS (ESI-):** Calculated mass for $C_{10}H_{11}ClN_4O_7P^-$:

365.0059 Da; found: $[M-H]^-$ = 365.0056 m/z;

**$^1$H-NMR:** (500 MHz, $D_2O$) δ (ppm) = 8.84 (s, 1H), 8.71 (s, 1H), 6.23 (d, J = 5.2 Hz, 1H), 4.78 (m, 1H, overlap with HOD), 4.54 (dd, J = 5.1, 4.1 Hz, 1H), 4.42 (dt, J = 5.2, 3.0 Hz, 1H), 4.20 (ddd, J = 11.7, 4.6, 2.9 Hz, 1H), 4.15 (ddd, J = 11.6, 5.5, 3.3 Hz, 1H).

**$^{13}$C-NMR:** (126 MHz, $D_2O$) δ (ppm) = 151.8, 151.1, 150.0, 145.2, 130.9, 88.0, 84.1, 74.5, 70.3, 64.2.

**$^{31}$P-NMR:** (202 MHz, $D_2O$) δ (ppm) = 0.49 (s).

## Polyphosphate kinase 2 from *Erysipelotrichaceae* bacterium (EbPPK2)

### Gene synthesis

pET28a(+)-EbPPK2-His$_6$ cloned with NcoI/XhoI restriction sites was ordered at BioCat (Heidelberg) as previously described.[1]

### DNA sequence

5′ATGGCAAATATCTACAAGATCGATAAGCTGAATAACTTTAACCTGAATAACCATAAGACC
GATGATTATAGTCTGTGTAAAGATAAAGACACCGCCCTGGAACTGACCCAGAAAAATATTCA
GAAAATCTATGACTACCAGCAGAAACTGTATGCCGAAAAGAAAGAAGGTCTGATTATTGCAT
TTCAGGCAATGGATGCAGCCGGCAAAGATGGCACCATTCGCGAAGTGCTGAAAGCACTGGCC
CCGCAGGGCGTTCATGAAAAACCGTTTAAAAGTCCGAGCAGTACCGAACTGGCACATGATTA
TCTGTGGCGCGTTCATAATGCAGTGCCGGAAAAAGGTGAAATTACCATTTTTAATCGCAGTC
ATTACGAAGATGTGCTGATTGGTAAAGTTAAAGAACTGTATAAGTTCCAGAACAAAGCCGAT
CGTATTGATGAAAATACCGTTGTGGATAATCGTTATGAAGATATTCGTAATTTCGAGAAATA
CCTGTATAACAATAGCGTTCGCATTATTAAGATCTTTCTGAATGTTAGTAAGAAGGAACAGG
CAGAACGCTTTCTGAGTCGTATTGAAGAACCGGAAAAGAATTGGAAATTTTCAGATAGCGAT
TTCGAAGAACGTGTTTATTGGGATAAATATCAGCAGGCATTTGAAGATGCCATTAATGCAAC
CAGTACCAAAGATTGCCCGTGGTATGTTGTGCCGGCAGATCGCAAATGGTATATGCGTTATG
TTGTTAGCGAAATTGTGGTTAAAACCCTGGAAGAAATGAATCCGAAATATCCGACCGTTACC
AAAGAAACCCTGGAACGTTTTGAAGGTTATCGTACCAAACTGCTGGAAGAATATAATTATGA
TCTGGATACCATCCGTCCGATTGAAAAACTCGAGCACCACCACCACCACCACTGA 3′

### Amino acid sequence

This construct (WT-EbPPK2-His$_6$): MW: 36.566 kDa, Molecular extinction coefficient: 57425 $M^{-1} \cdot cm^{-1}$

MANIYKIDKLNNFNLNNHKTDDYSLCKDKDTALELTQKNIQKIYDYQQKLYAEKKEGLIIAF
QAMDAAGKDGTIREVLKALAPQGVHEKPFKSPSSTELAHDYLWRVHNAVPEKGEITIFNRSH
YEDVLIGKVKELYKFQNKADRIDENTVVDNRYEDIRNFEKYLYNNSVRIIKIFLNVSKKEQA
ERFLSRIEEPEKNWKFSDSDFEERVYWDKYQQAFEDAINATSTKDCPWYVVPADRKWYMRYV
VSEIVVKTLEEMNPKYPTVTKETLERFEGYRTKLLEEYNYDLDTIRPIEKLEHHHHHH

## Site-directed mutagenesis

Site-directed mutagenesis was performed using a previously described protocol.[2]

Point mutations were introduced by PCR using the pET28a(+)-EbPPK2 plasmid as a template. Each reaction contained either reverse or forward primer (4 µL of each 10 µM stock, Biolegio), DNA template (0.5 µL, 50-100 ng), dNTPs (1 µL, 25 mM, Solis Biodyne), Phusion High-Fidelity DNA Polymerase (1 µL, 2 U/µL stock, ThermoScientific), HF buffer (10 µL, 5X, ThermoScientific) and doubly distilled water (33.5 µL). The PCR protocol consisted of an initial step of 98 °C for 1 min, followed by 10 cycles of 98 °C for 30 sec, 55 °C for 1 min, 72 °C for 3.5 min and then a final step of 72 °C for 10 min. Afterwards both reactions (forward and reverse) were combined, Phusion High-Fidelity DNA Polymerase (0.75 µL, 2 U/µL stock, ThermoScientific) was added and the PCR protocol was performed for another 18 cycles. The template DNA was digested with FastDigest DpnI (1 µL, ThermoScientific) for 1 h at 37 °C, followed by 5 min at 80 °C. The resulting mixture was purified using the NucleoSpin® Gel and PCR Clean-up (Macherey-Nagel) according to manufacturer's procedures, then used for transformation of electrocompetent *E. coli* TOP10 cells. The sequence was verified by DNA sequencing (Microsynth).

## Primers used for site-directed mutagenesis

| Variant | Sequence | Parent plasmid |
|---|---|---|
| D127A | 5′-ttttaatcgcagtcattacgaagctgtgctgattggtaaagttaaag-3′<br>5′-ctttaactttaccaatcagcacagcttcgtaatgactgcgattaaaa-3′ | pET28a(+)-EbPPK2 |
| D127N | 5′-attaccatttttaatcgcagtcattacgaaaatgtgctgattggtaaa-3′<br>5′-tttaccaatcagcacattttcgtaatgactgcgattaaaaatggtaat-3′ | pET28a(+)-EbPPK2 |

## EbPPK2 D127A

MANIYKIDKLNNFNLNNHKTDDYSLCKDKDTALELTQKNIQKIYDYQQKLYAEKKEGLIIAF

QAMDAAGKDGTIREVLKALAPQGVHEKPFKSPSSTELAHDYLWRVHNAVPEKGEITIFNRSH

YE**A**VLIGKVKELYKFQNKADRIDENTVVDNRYEDIRNFEKYLYNNSVRIIKIFLNVSKKEQA

ERFLSRIEEPEKNWKFSDSDFEERVYWDKYQQAFEDAINATSTKDCPWYVVPADRKWYMRYV

VSEIVVKTLEEMNPKYPTVTKETLERFEGYRTKLLEEYNYDLDTIRPIEKLEHHHHHH

## EbPPK2 D127N

MANIYKIDKLNNFNLNNHKTDDYSLCKDKDTALELTQKNIQKIYDYQQKLYAEKKEGLIIAF

QAMDAAGKDGTIREVLKALAPQGVHEKPFKSPSSTELAHDYLWRVHNAVPEKGEITIFNRSH

YE**N**VLIGKVKELYKFQNKADRIDENTVVDNRYEDIRNFEKYLYNNSVRIIKIFLNVSKKEQA

ERFLSRIEEPEKNWKFSDSDFEERVYWDKYQQAFEDAINATSTKDCPWYVVPADRKWYMRYV

VSEIVVKTLEEMNPKYPTVTKETLERFEGYRTKLLEEYNYDLDTIRPIEKLEHHHHHH

**EbPPK2 expression and purification**

Wildtype EbPPK2 (WT-EbPPK2) was expressed and purified as previously described and yielded 125 mg of soluble protein per litre of culture.[1]

For EbPPK2-variant production, *E. coli* BL21(DE3) cells were transformed with either pET28a(+)-EbPPK2-D127A-$His_6$ or pET28a(+)-EbPPK2-D127N-$His_6$ by heat shock at 42 °C for 1 min, regenerated in LB media for 1 h at 37 °C and 650 rpm, cultivated in 15 mL in LB medium with kanamycin (50 µg/mL) at 37 °C and 180 rpm for 16 h. 15 mL overnight culture were added to 1 L of LB medium with kanamycin and grown to an $OD_{600}$ = 0.8. Expression was induced with 0.5 mM IPTG at 20 °C for 16 hours. Cells were harvested by centrifugation (5000 xg, 4 °C, 30 min) and pellets were stored at -80 °C.

Pellets were resuspended in Buffer A (50 mM Tris-HCl, pH 7.5, 300 mM NaCl, 10 mM imidazole, 10 % glycerol, filtered, degassed) and lysed by sonication (60 % amplitude, 1 second on/off pulses for 5 min, 2 times) by a Sonopuls GM3100 (Bandelin). Cell debris was removed by centrifugation (11000 xg, 4 °C, 30 min) and the supernatant was filtered through a 0.2 µm syringe filter and loaded into a Superloop™. Protein purification was performed by IMAC using a HisTrap FF 5 mL (GE Healthcare) column connected to a ÄKTA Purifier system (GE Healthcare). The protein was eluted with Buffer B (50 mM Tris pH 7.5, 300 mM NaCl, 500 mM imidazole, 10 % glycerol). Fractions containing EbPPK2 were concentrated, and the buffer was exchanged to Buffer C (50 mM Tris pH 7.5, 300 mM NaCl, 10 % glycerol) using Amicon Ultra-15 centrifuge filters (UFC903024, 30 kDa cut off). The concentration was determined by absorbance at 280 nm. Aliquots (100 µM) were flash frozen in liquid nitrogen and stored at -80 °C.

Purifications of EbPPK2 D127A yielded 110 mg and EbPPK2 D127N 90 mg of soluble protein per litre of culture.

**EbPPK2 reactions**

For EbPPK2 reactions, 1 mM NMP (**1a-10a**) was incubated in 20 mM Tris (pH 8) with 20 mM $MgCl_2$, 6.1 g/L polyphosphate and 1 µM EbPPK2 in a total volume of 40 µL at 30 °C. Samples were taken after indicated timepoints, the enzyme was denatured at 80 °C for 2 min, and the samples were centrifuged (10 minutes, 21000x g, 4 °C) to remove precipitated protein. The supernatant (1 µL) was analysed *via* HPLC methods A or B.

Note: Reactions containing 6-thioguanosine nucleotides were diluted 10-fold in water and 1 µL was injected into the HPLC.

**Protein crystallization**

EbPPK2 was further purified by size exclusion chromatography in buffer D (25 mM HEPES pH 7.5, 300 mM NaCl, 1 mM $MgCl_2$, 1 mM TCEP). 10 mg/mL of protein was mixed with 1.5 mM adenosine-5′-[(β,γ)-methyleno]triphosphate (ACP) and 1.2 mg/mL polyphosphate (polyP). Crystals were grown by mixing of protein and reservoir solution with an ARI Gryphon robot to Swissci MRC2 well plates and incubated at 12 °C in a sitting drop vapor diffusion setup. Crystals containing ACP and polyP grew with 25 % MPD, 0.1 M sodium acetate, 0.2 M sodium chloride, pH 4.6 as precipitant solution. Crystals of EbPPK2 with polyP grew with 10 % MPD, 0.1 M sodium acetate, 0.2 M sodium chloride, pH 5.4 reservoir solution. Crystals were harvested and directly flash cooled in liquid nitrogen. Diffraction data were collected at DESY/PETRA III beamline P13[3]. Raw data were indexed, integrated and scaled in XDS[4-5]. The CCP4 suite[6] running on the ccp4 cloud servers[7] was used for structure solution and refinement. For molecular replacement, a single chain ensemble search model was generated from the truncated top five hits of an AlphaFold2 modelling.[8] Molecular replacement was performed in phaser[9]. The initial model was manually curated in Coot 0.9.8.95 and refined using refmac[10]. Ligand restraints were created in AceDRG[11]. Structures were analyzed and depicted in Chimera X 1.8[12]. To reveal the ligand binding position, a polder map was calculated with the phenix software suite. The polyphosphate and ACP ligand were omitted from density calculations.[13-14]

# Supplementary figures and tables

## Synthesis of 6-chloropurine riboside 5′-monophosphate (10a)

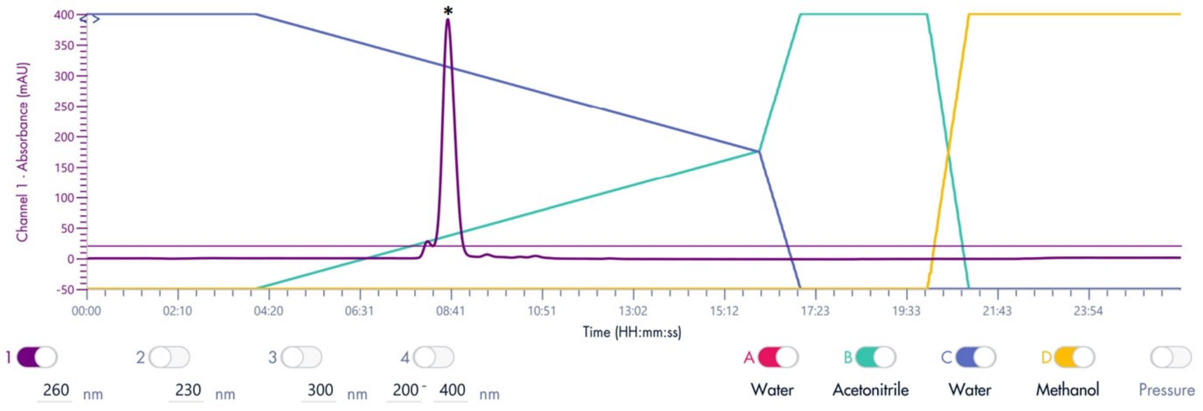


Fig. S1: C18-flash purification of 6-chloropurine riboside 5′-monophosphate (**10a**). The fraction marked with * was collected.

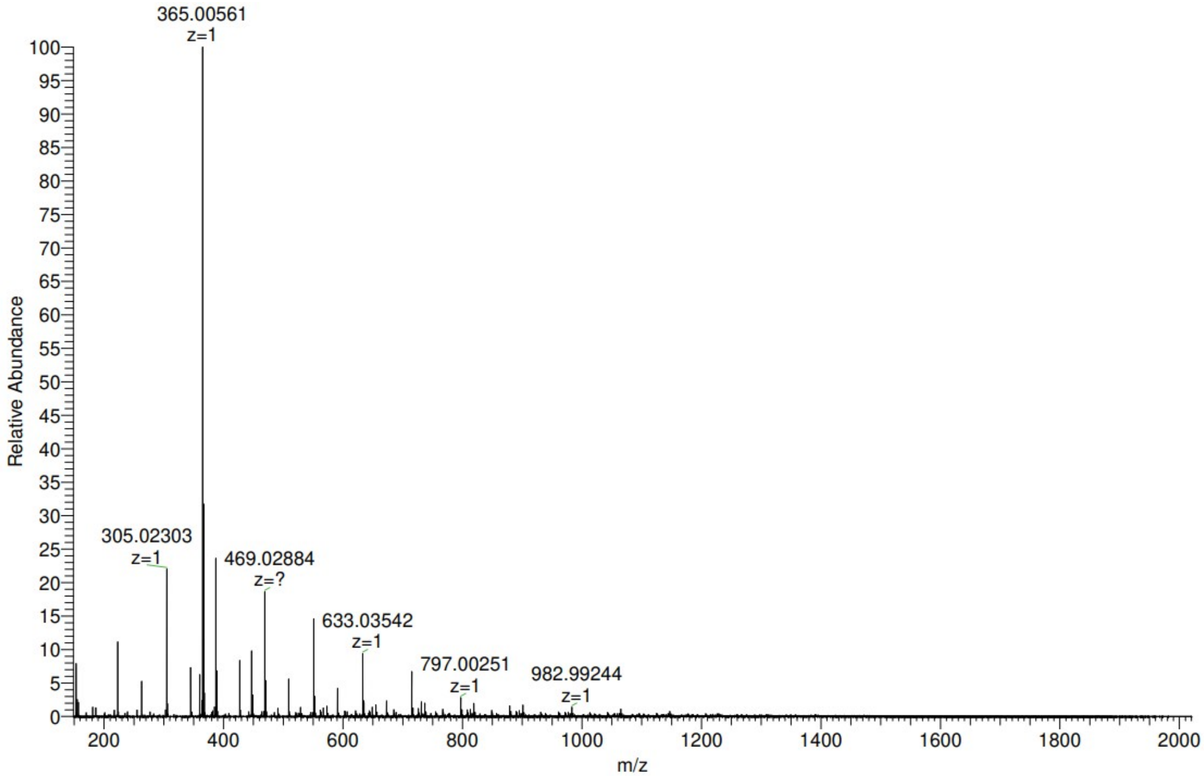


Fig. S2: HRMS of 6-chloropurine riboside 5′-monophosphate (**10a**). Calculated mass for $C_{10}H_{11}ClN_4O_7P^-$: 365.0059 Da; found: $[M\text{-}H]^-$ = 365.0056 m/z.

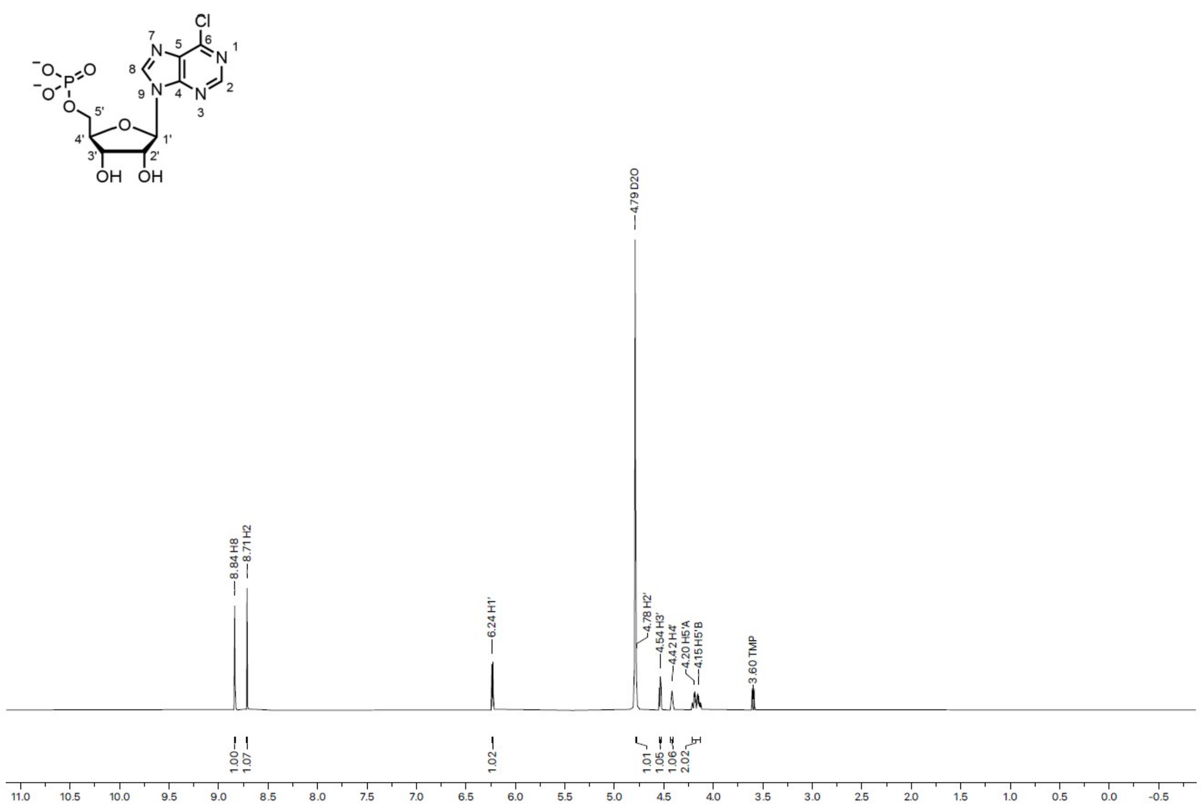


Fig. S3: $^{1}$H-NMR of 6-chloropurine riboside 5′-monophosphate (**10a**).

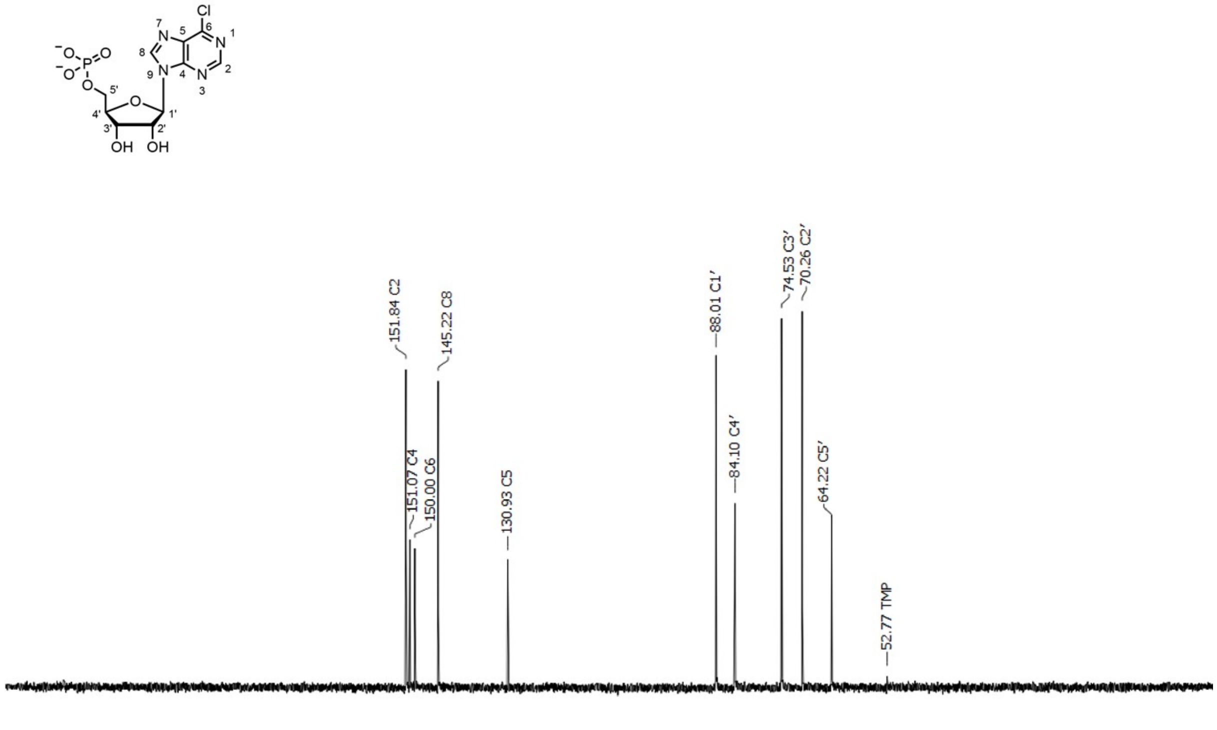


Fig. S4: $^{13}$C-NMR of 6-chloropurine riboside 5′-monophosphate (**10a**).

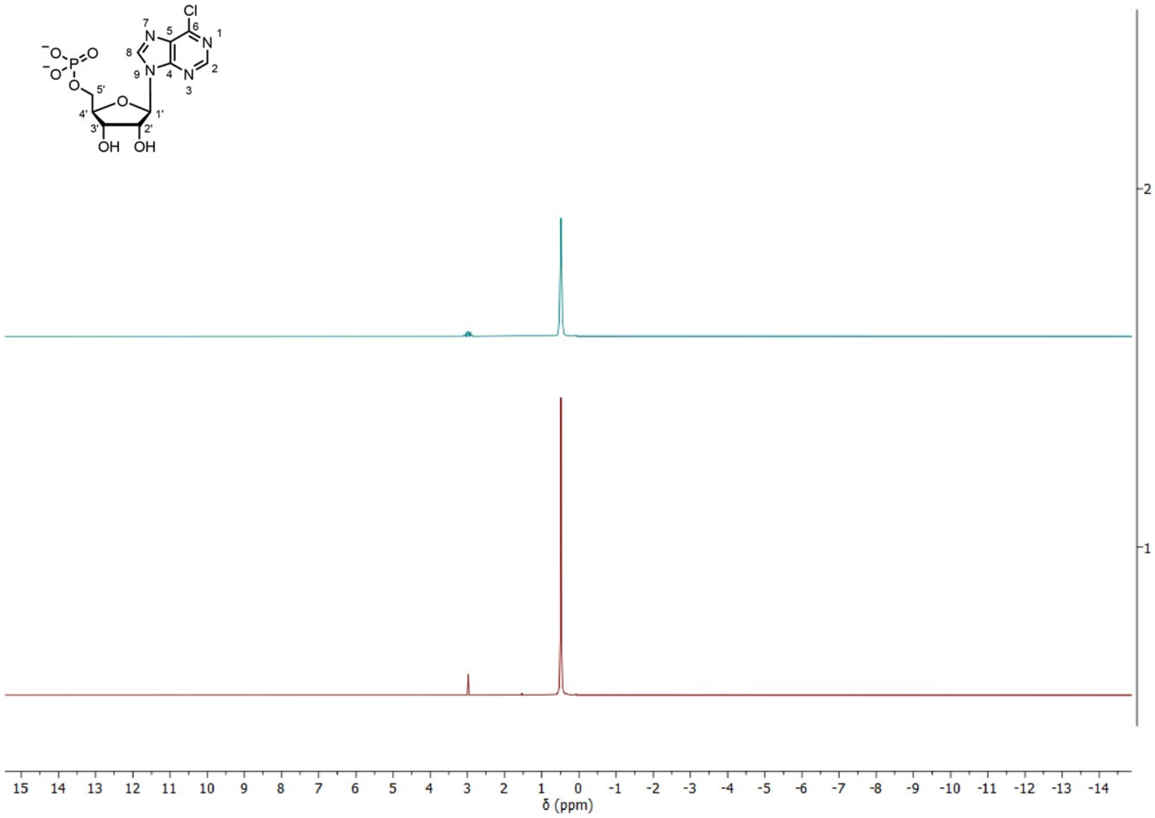


Fig. S5: $^{31}$P-NMR of 6-chloropurine riboside 5′-monophosphate (**10a**). Top panel without proton decoupling, bottom panel without decoupling.

## HPLC analysis of EbPPK2-catalysed reactions

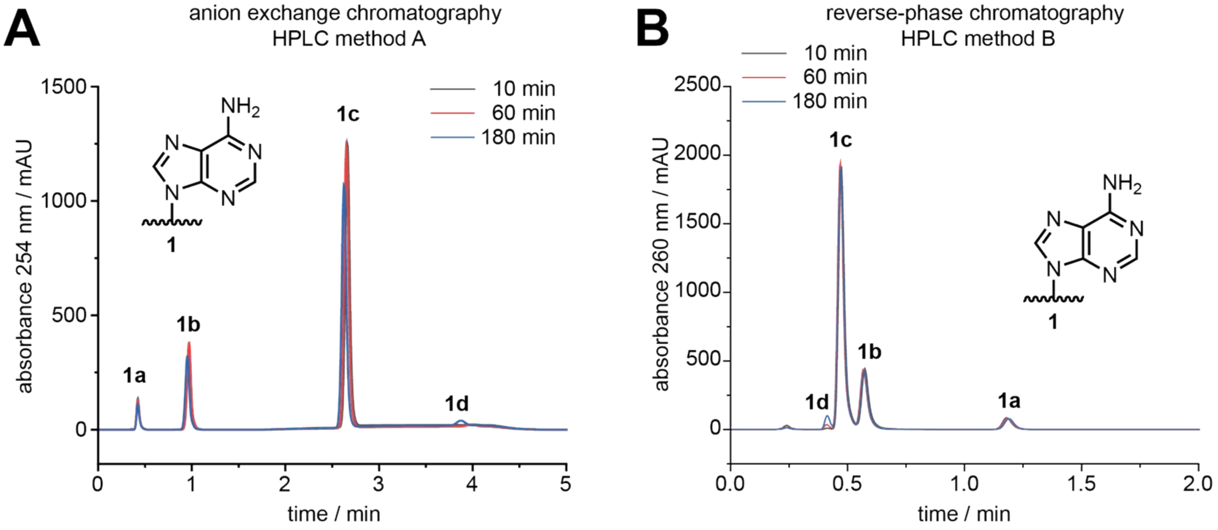


Fig. S6: Representative HPLC analysis of the reaction of EbPPK2 starting from AMP (**1a**). A) HPLC method A (anion exchange) results in the order of elution AMP (**1a**), ADP (**1b**), ATP (**1c**) and A4P (**1d**). B) HPLC method B ($C_{18}$) results in the reversed order of elution.

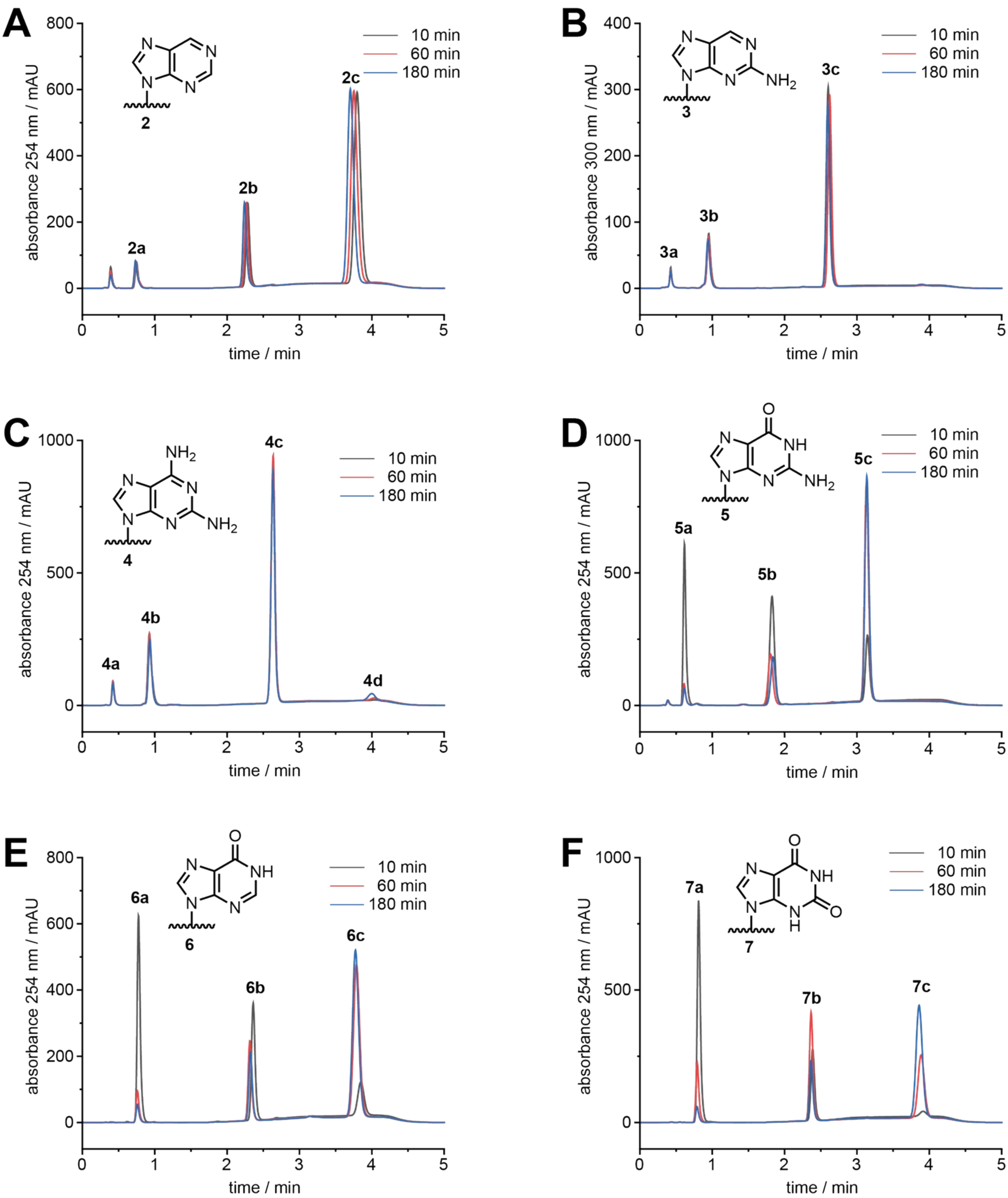


Fig. S7: A)-F) Representative HPLC analysis (method A) of the reaction of EbPPK2 starting from indicated AMP or GMP analogues (**2a-7a**).

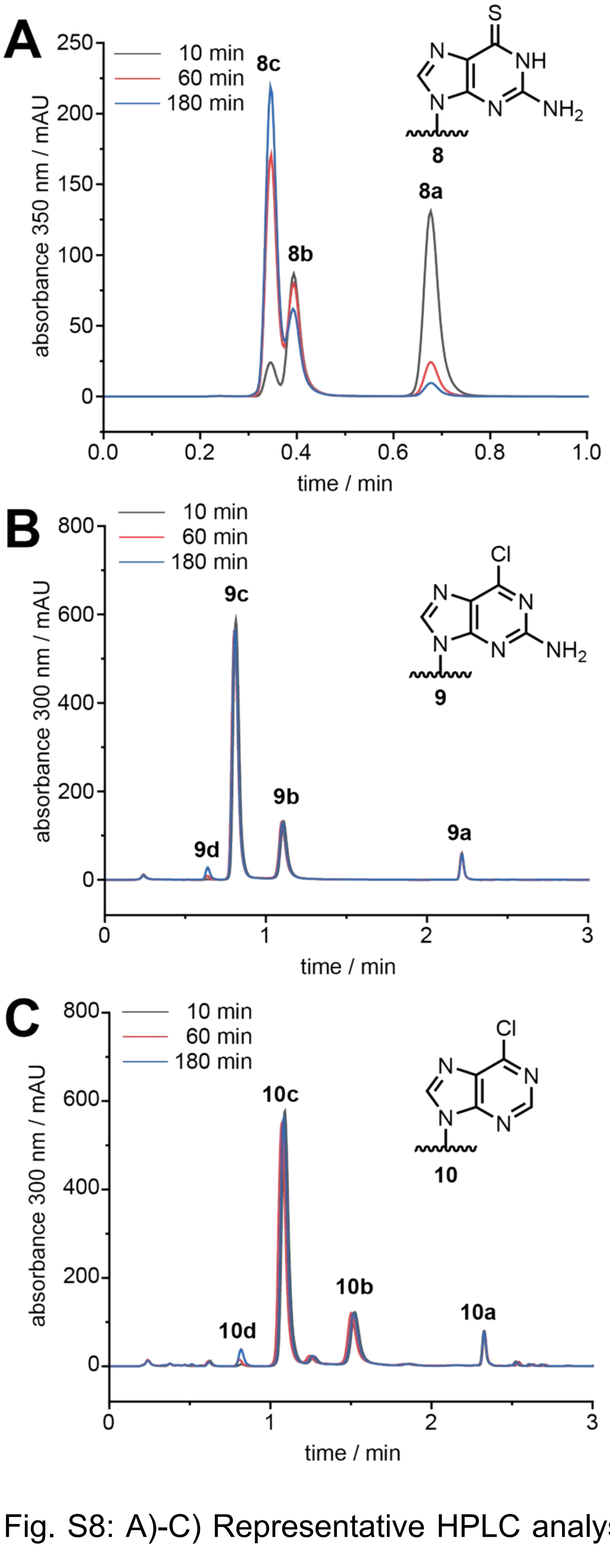


Fig. S8: A)-C) Representative HPLC analysis (method B) of the reaction of EbPPK2 starting from indicated AMP analogues (**8a-10a**).

**Table S1: Conversions of EbPPK2 reactions, starting from 1a-10a.** Average conversion and standard deviation of three independent experiments. Related to figure 2.

| | **d** (N4P) | | **c** (NTP) | | **b** (NDP) | | **a** (NMP) | |
|---|---|---|---|---|---|---|---|---|
| **10 min** | conv. (%) | ± SD | conv. (%) | ± SD | conv. (%) | ± SD | conv. (%) | ± SD |
| | | | | | | | | |
| **1** | 0,2 | 0,17 | 74,7 | 0,35 | 19,8 | 0,10 | 5,3 | 0,15 |
| **2** | | | 76,2 | 0,35 | 18,7 | 0,20 | 5,1 | 0,17 |
| **3** | 0,1 | 0,19 | 73,6 | 0,51 | 20,8 | 0,29 | 5,5 | 0,18 |
| **4** | 0,2 | 0,13 | 72,7 | 0,34 | 22,5 | 0,23 | 4,7 | 0,04 |
| **5** | | | 23,0 | 1,27 | 41,9 | 0,44 | 35,1 | 1,03 |
| **6** | | | 16,8 | 0,33 | 33,2 | 0,23 | 49,9 | 0,40 |
| **7** | | | 3,6 | 0,29 | 27,0 | 0,52 | 69,3 | 0,81 |
| **8** | | | 6,8 | 0,32 | 33,4 | 0,17 | 59,8 | 0,43 |
| **9** | 0,1 | 0,01 | 73,3 | 0,0009 | 21,5 | 0,09 | 5,1 | 0,11 |
| **10** | 0,1 | 0,15 | 72,7 | 0,31 | 21,4 | 0,09 | 5,7 | 0,07 |

| | **d** (N4P) | | **c** (NTP) | | **b** (NDP) | | **a** (NMP) | |
|---|---|---|---|---|---|---|---|---|
| **60 min** | conv. (%) | ± SD | conv. (%) | ± SD | conv. (%) | ± SD | conv. (%) | ± SD |
| | | | | | | | | |
| **1** | 1,2 | 0,19 | 74,3 | 0,25 | 19,5 | 0,08 | 5,0 | 0,05 |
| **2** | | | 75,6 | 0,21 | 18,8 | 0,16 | 5,6 | 0,23 |
| **3** | 0,6 | 0,17 | 73,6 | 0,41 | 20,5 | 0,10 | 5,3 | 0,19 |
| **4** | 1,5 | 0,27 | 73,1 | 0,37 | 20,8 | 0,42 | 4,6 | 0,04 |
| **5** | | | 75,2 | 0,38 | 20,0 | 0,30 | 4,8 | 0,10 |
| **6** | | | 71,9 | 0,71 | 21,1 | 0,45 | 7,0 | 0,27 |
| **7** | | | 40,7 | 1,24 | 39,9 | 0,48 | 19,4 | 0,87 |
| **8** | | | 53,7 | 0,40 | 34,4 | 0,39 | 11,9 | 0,10 |
| **9** | 0,7 | 0,04 | 72,8 | 0,03 | 21,4 | 0,10 | 5,1 | 0,11 |
| **10** | 0,9 | 0,12 | 72,9 | 1,36 | 20,8 | 0,84 | 5,4 | 0,40 |

| | **d** (N4P) | | **c** (NTP) | | **b** (NDP) | | **a** (NMP) | |
|---|---|---|---|---|---|---|---|---|
| **180 min** | conv. (%) | ± SD | conv. (%) | ± SD | conv. (%) | ± SD | conv. (%) | ± SD |
| | | | | | | | | |
| **1** | 2,9 | 0,22 | 72,9 | 0,23 | 19,2 | 0,06 | 4,9 | 0,08 |
| **2** | | | 75,1 | 0,30 | 19,3 | 0,25 | 5,6 | 0,09 |
| **3** | 1,4 | 0,29 | 73,5 | 0,54 | 20,0 | 0,13 | 5,1 | 0,15 |
| **4** | 4,2 | 0,41 | 71,8 | 0,19 | 19,5 | 0,33 | 4,5 | 0,07 |
| **5** | | | 77,4 | 0,14 | 18,6 | 0,16 | 4,0 | 0,06 |
| **6** | | | 77,1 | 0,22 | 18,6 | 0,14 | 4,3 | 0,13 |
| **7** | | | 72,9 | 0,26 | 21,9 | 0,15 | 5,2 | 0,16 |
| **8** | | | 70,6 | 0,44 | 24,9 | 0,25 | 4,4 | 0,69 |
| **9** | 2,2 | 0,12 | 71,5 | 0,21 | 21,2 | 0,03 | 5,1 | 0,06 |
| **10** | 2,9 | 0,17 | 70,6 | 0,34 | 21,0 | 0,08 | 5,5 | 0,08 |

**LC-TOF-MS analysis of EbPPK2-catalysed reactions**

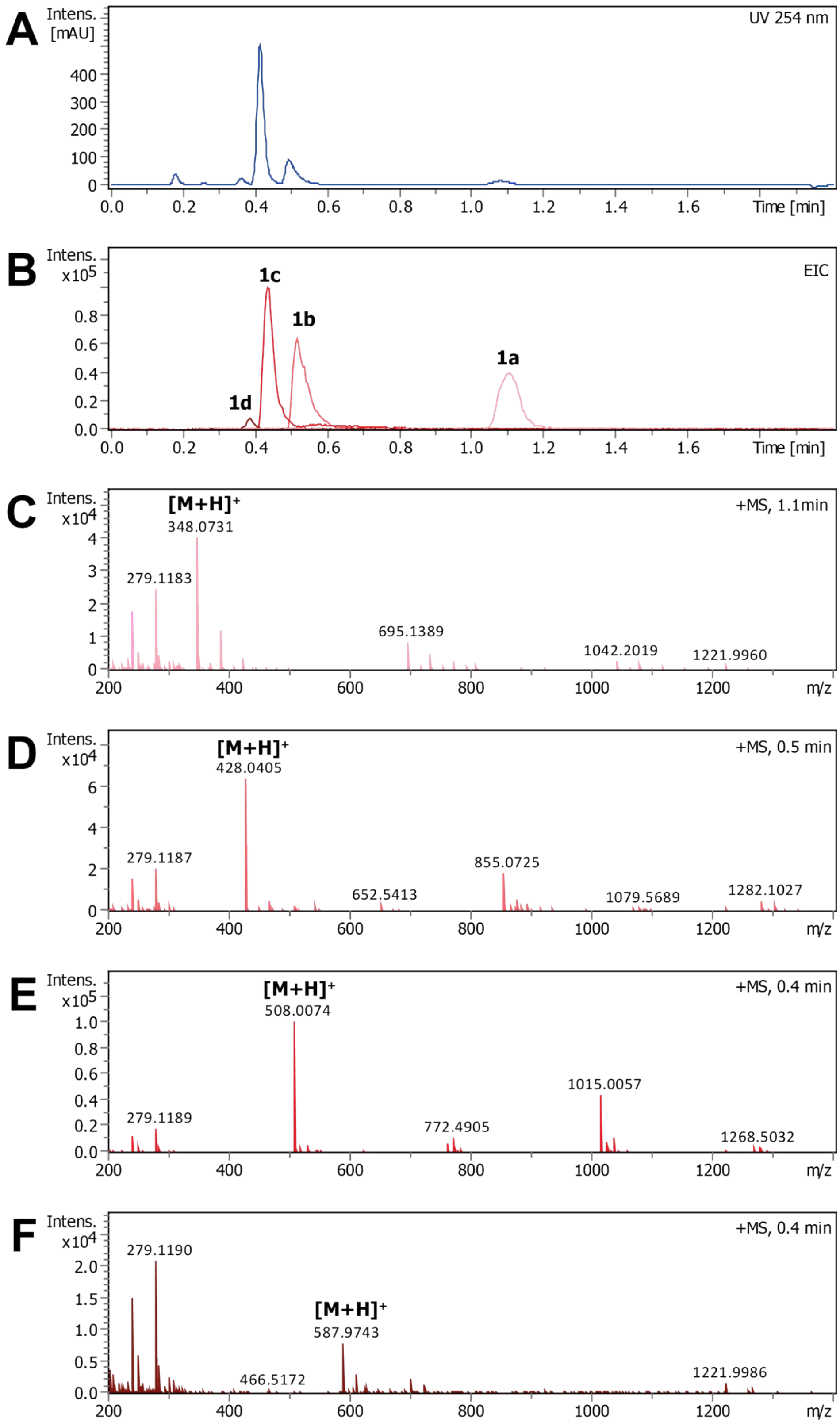


Fig. S9: LC-TOF-MS analysis of the reaction of EbPPK2 starting from **1a**. A) UV chromatogram at 254 nm. B) Extracted-ion chromatogram for **1a**-**1d**. C)-F) Mass spectrum for **1a**-**1d**.

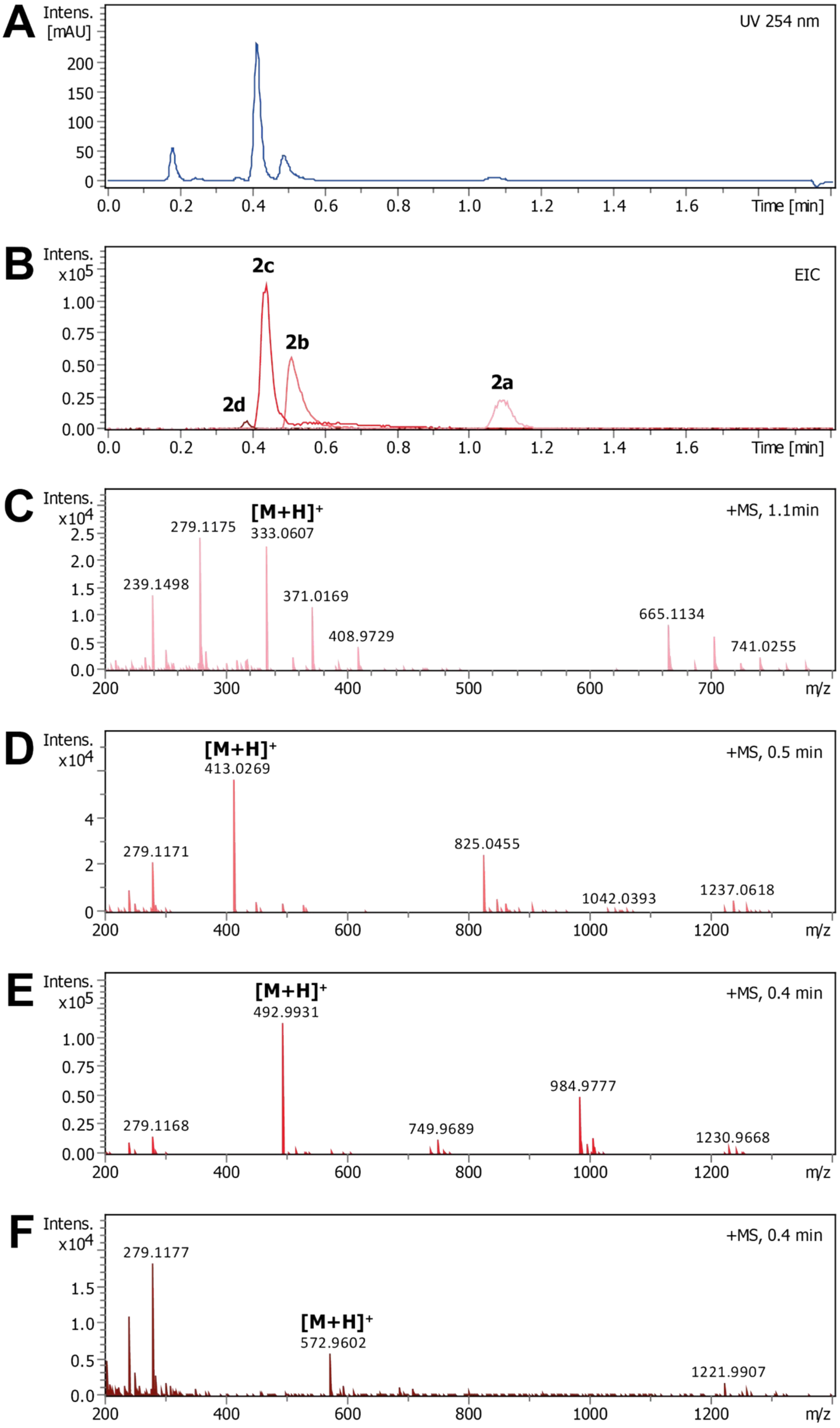


Fig. S10: LC-TOF-MS analysis of the reaction of EbPPK2 starting from **2a**. A) UV chromatogram at 254 nm. B) Extracted-ion chromatogram for **2a**-**2d**. C)-F) Mass spectrum for **2a**-**2d**.

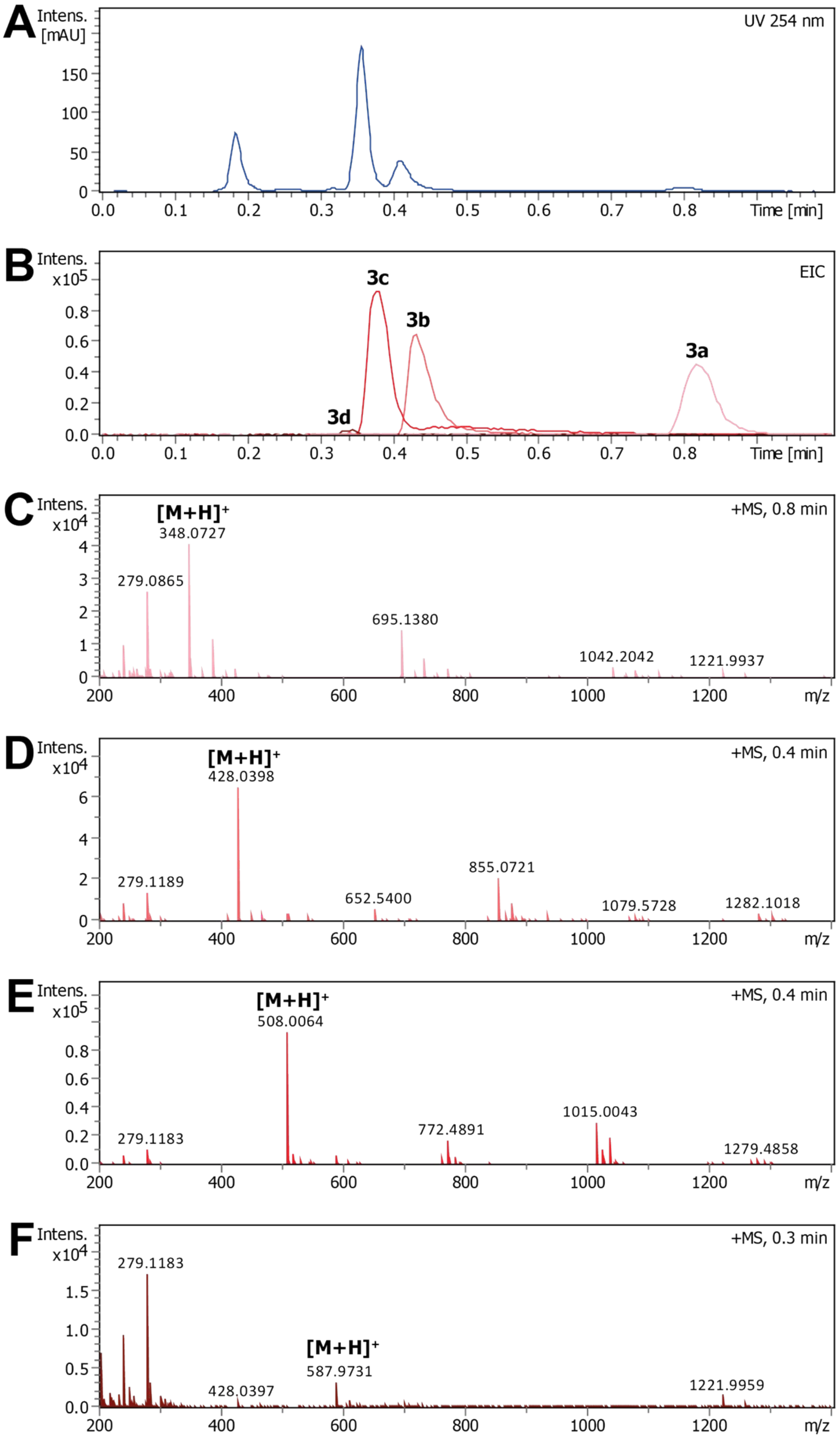


Fig. S11: LC-TOF-MS analysis of the reaction of EbPPK2 starting from **3a**. A) UV chromatogram at 254 nm. B) Extracted-ion chromatogram for **3a**-**3d**. C)-F) Mass spectrum for **3a**-**3d**.

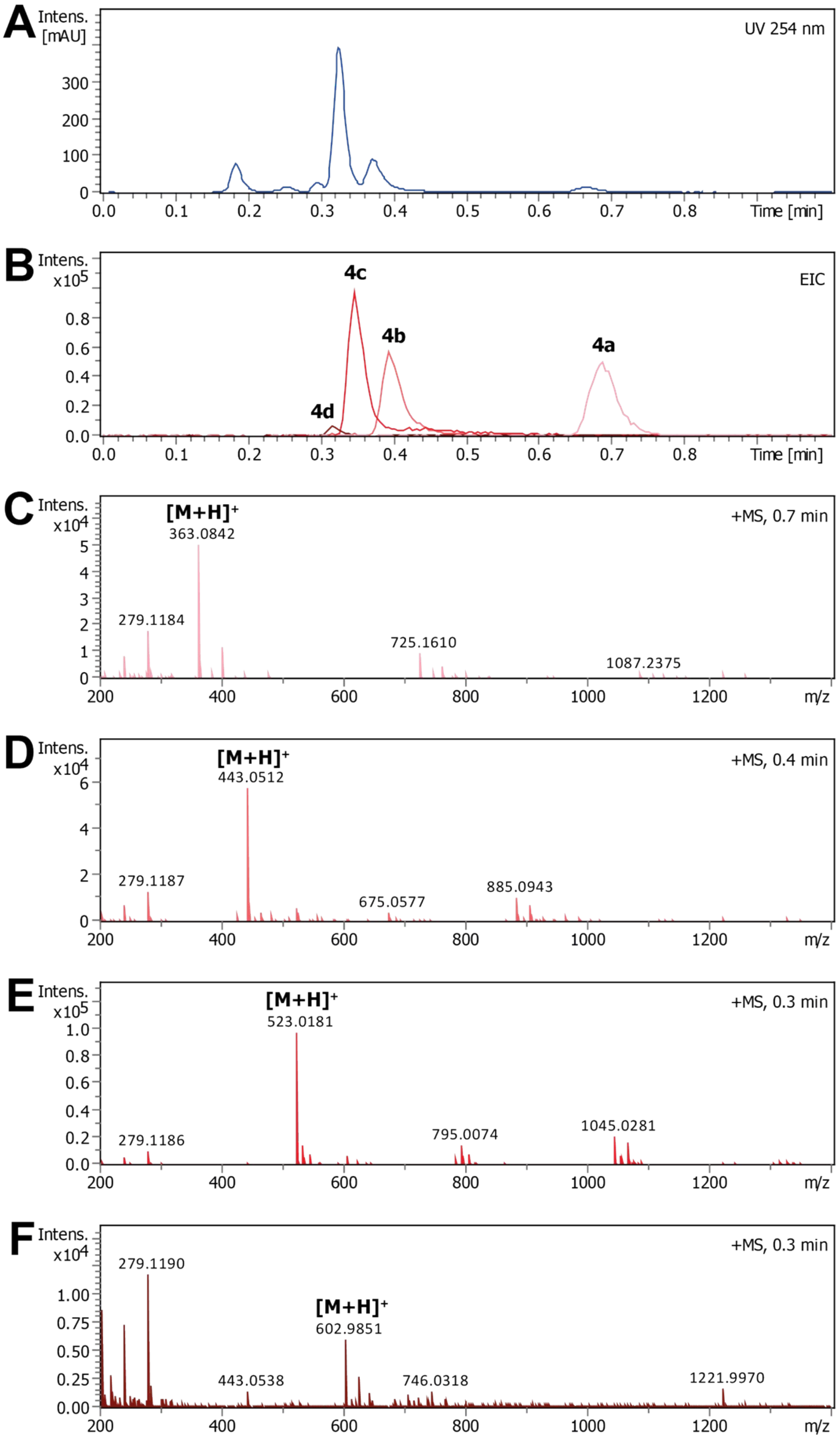


Fig. S12: LC-TOF-MS analysis of the reaction of EbPPK2 starting from **4a**. A) UV chromatogram at 254 nm. B) Extracted-ion chromatogram for **4a**-**4d**. C)-F) Mass spectrum for **4a**-**4d**.

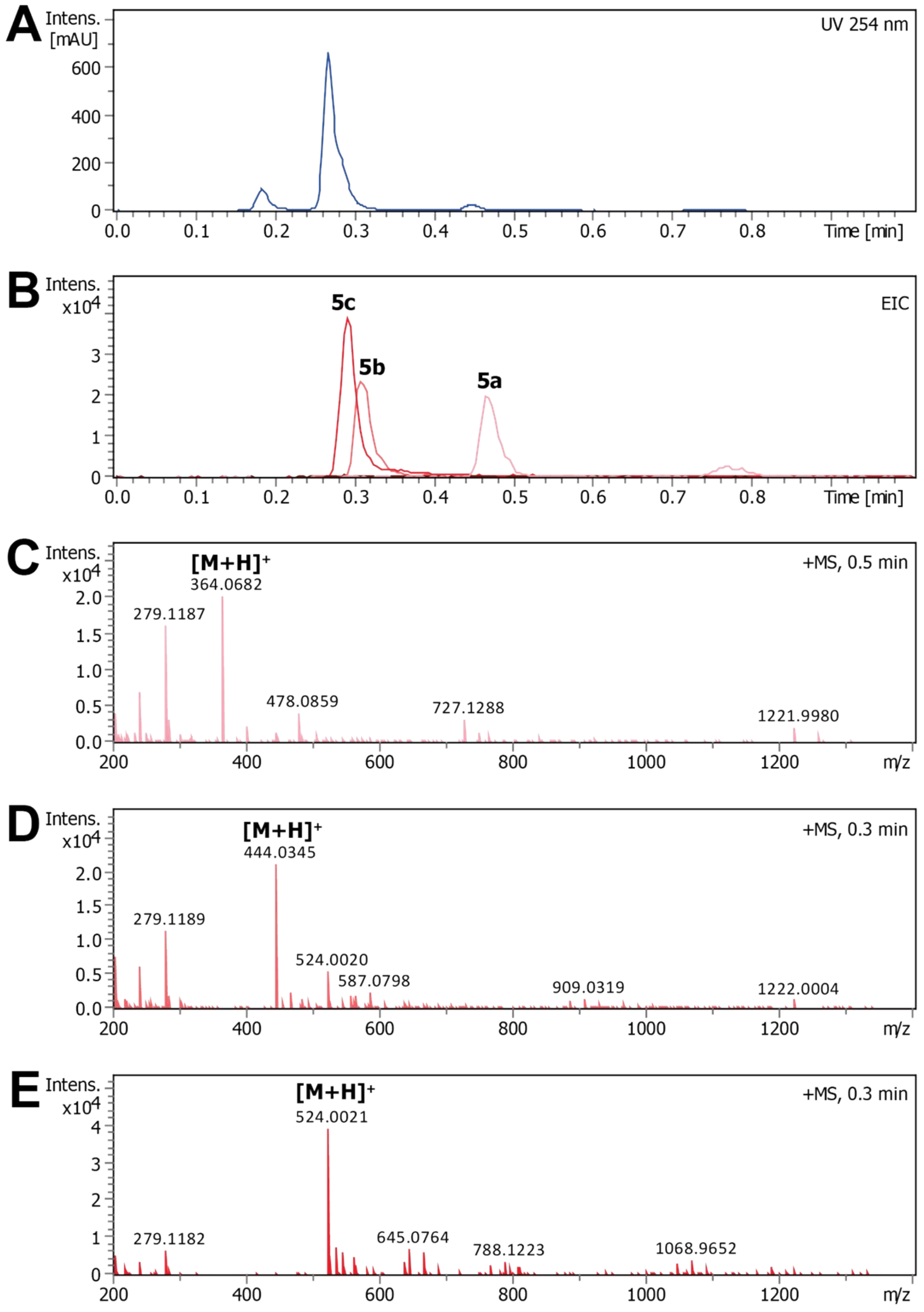


Fig. S13: LC-TOF-MS analysis of the reaction of EbPPK2 starting from **5a**. A) UV chromatogram at 254 nm. B) Extracted-ion chromatogram for **5a**-**5c**. C)-E) Mass spectrum for **5a**-**5c**.

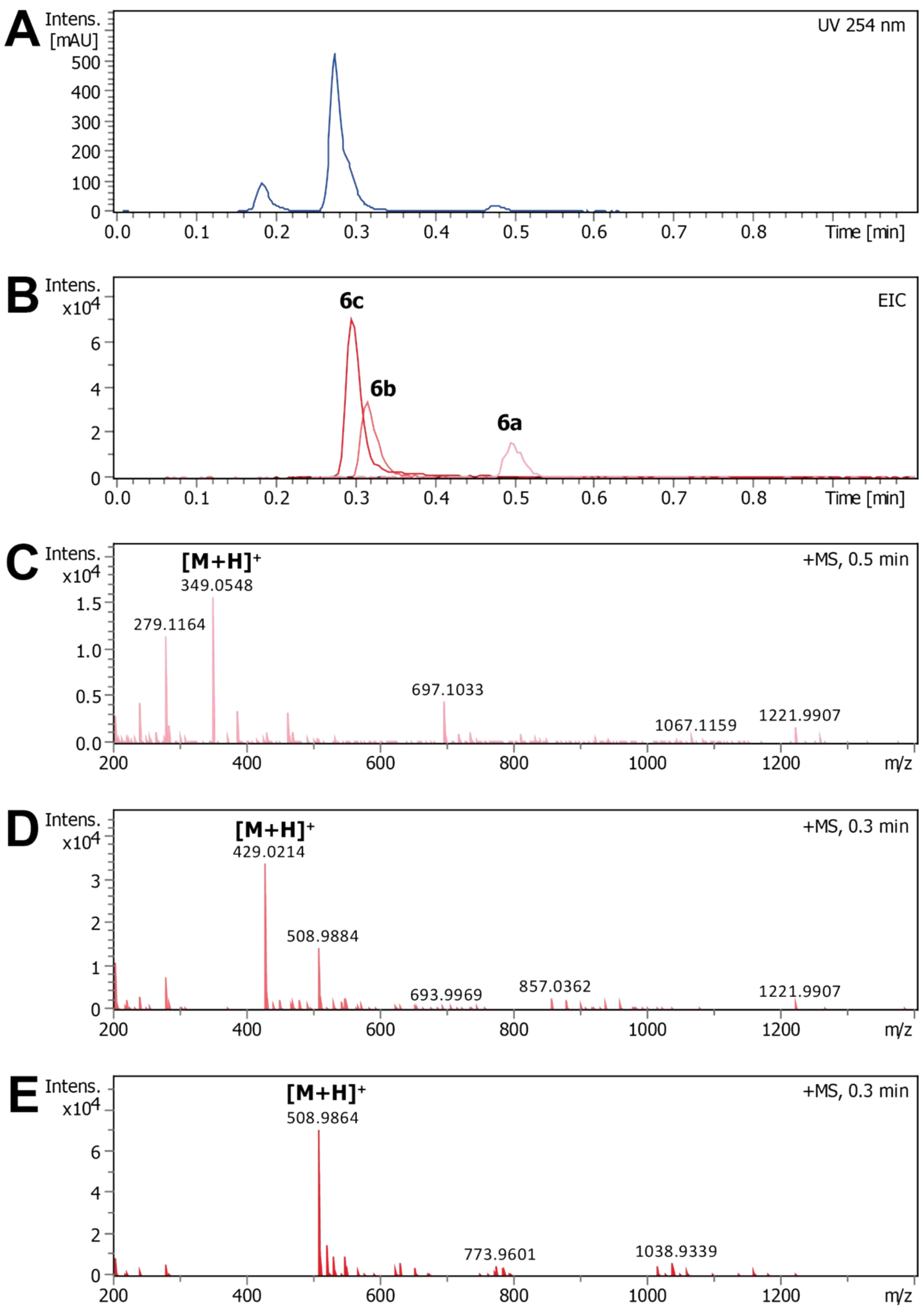


Fig. S14: LC-TOF-MS analysis of the reaction of EbPPK2 starting from **6a**. A) UV chromatogram at 254 nm. B) Extracted-ion chromatogram for **6a**-**6c**. C)-E) Mass spectrum for **6a-6c**.

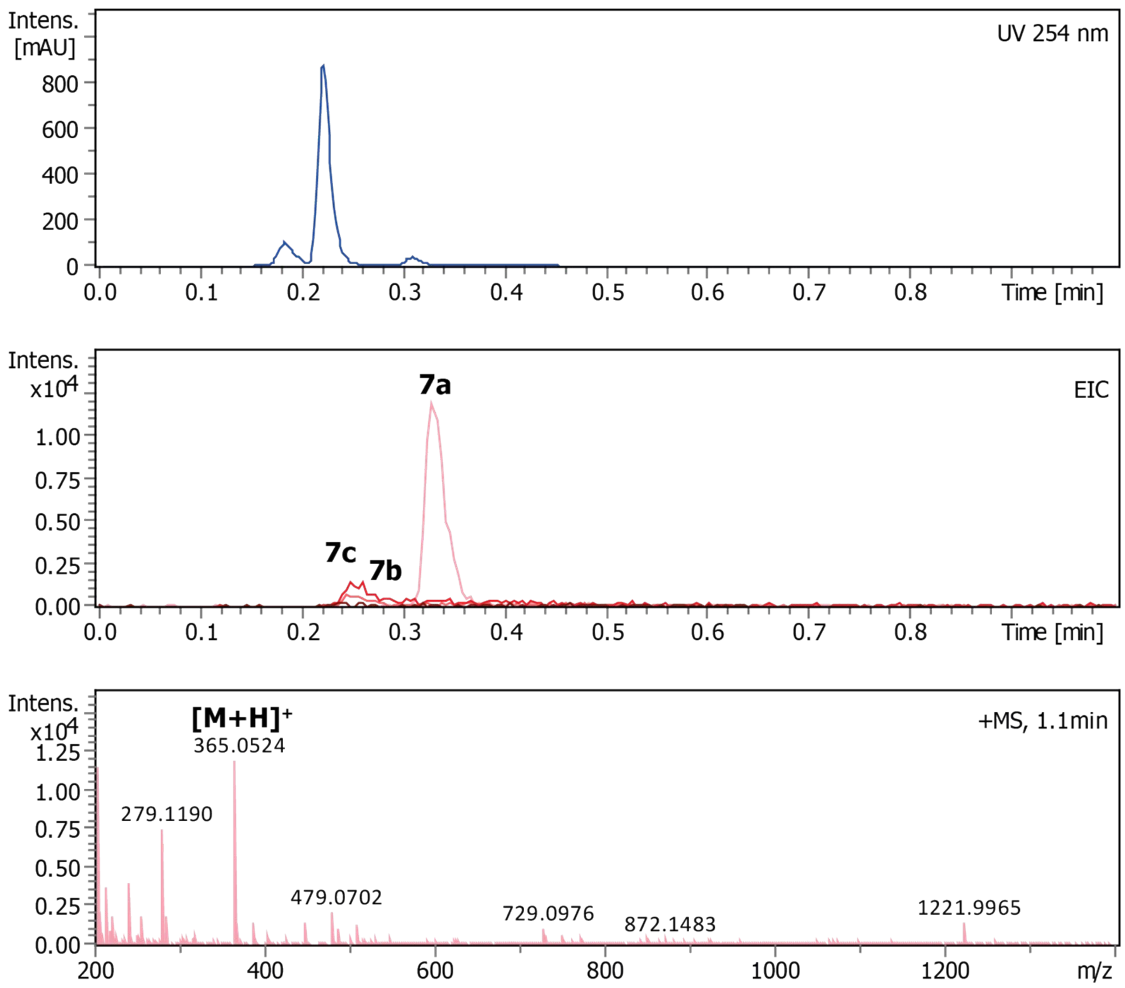


Fig. S15: LC-TOF-MS analysis of the reaction of EbPPK2 starting from **7a**. A) UV chromatogram at 254 nm. B) Extracted-ion chromatogram for **7a**-**7c**. C) Mass spectrum for **7a**.

Note that the UV chromatogram clearly shows the formation of higher phosphorylated species, while the mass signal for **7b** and **7c** is barely detectable. This loss of intensity might result from oxidation processes reported for xanthine[15] or low ionisation in ESI-positive mode

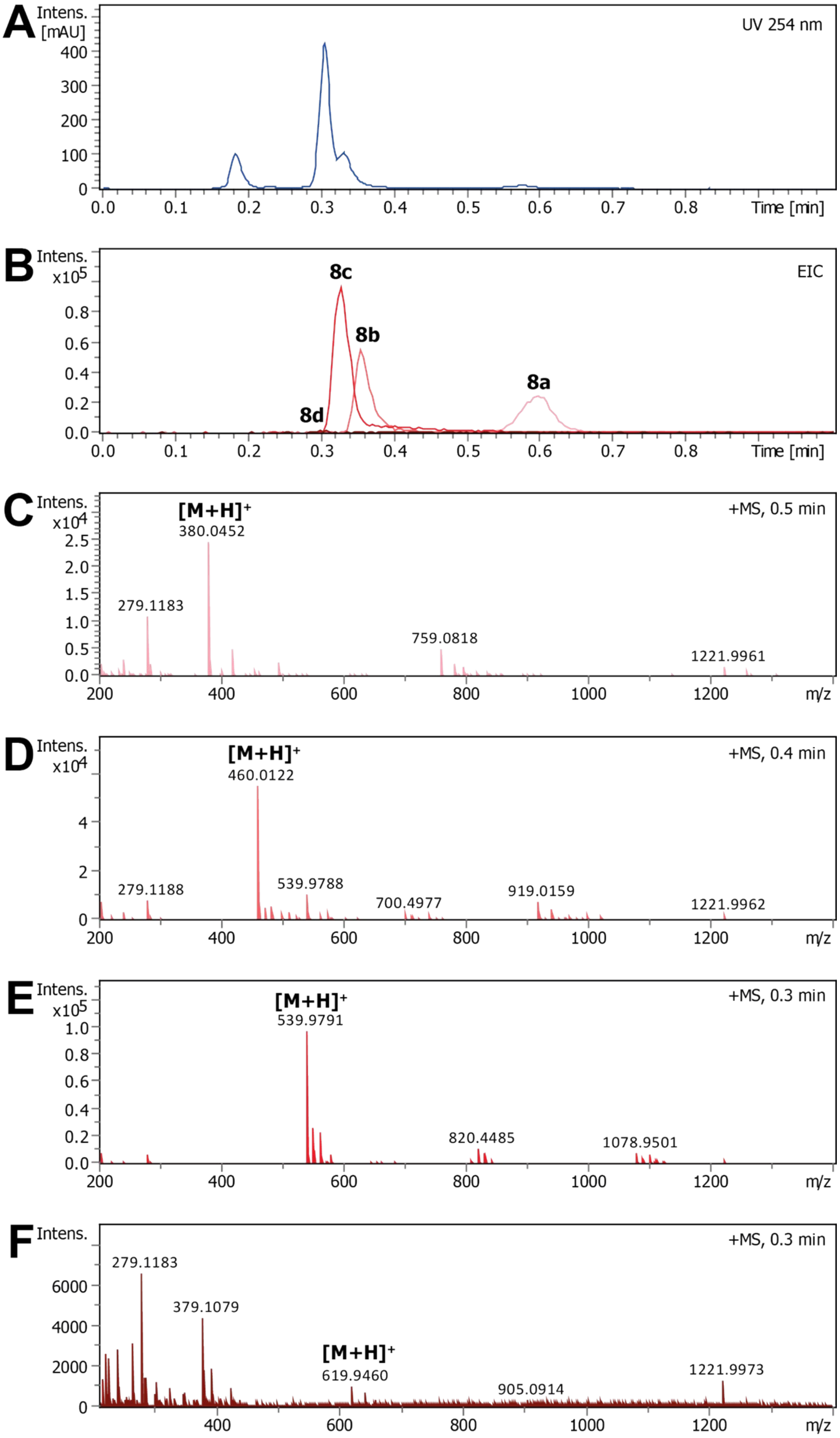


Fig. S16: LC-TOF-MS analysis of the reaction of EbPPK2 starting from **8a**. A) UV chromatogram at 254 nm. B) Extracted-ion chromatogram for **8a**-**8d**. C)-F) Mass spectrum for **8a**-**8d**.

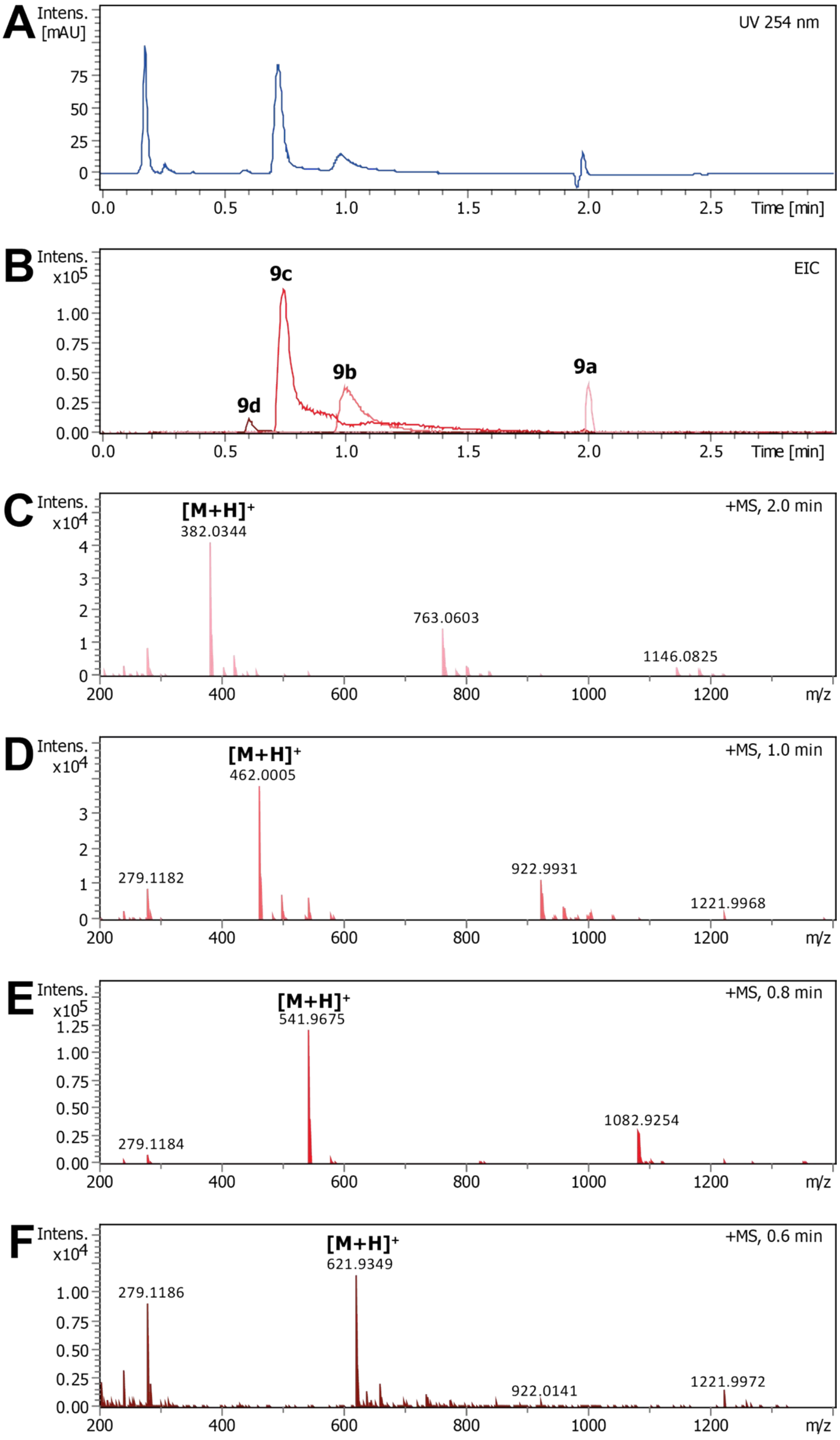


Fig. S17: LC-TOF-MS analysis of the reaction of EbPPK2 starting from **9a**. A) UV chromatogram at 254 nm. B) Extracted-ion chromatogram for **9a**-**9d**. C)-F) Mass spectrum for **9a**-**9d**.

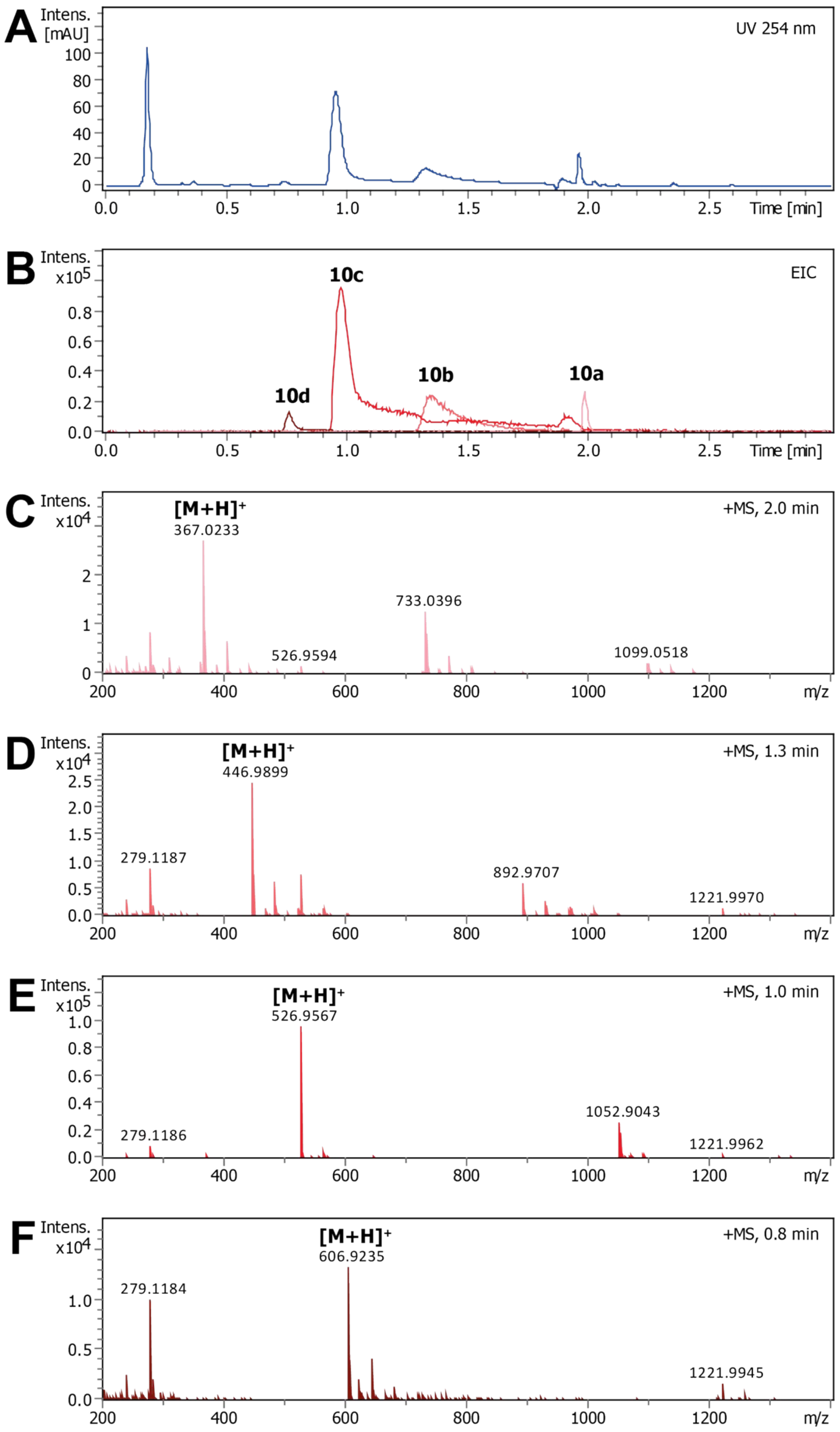


Fig. S18: LC-TOF-MS analysis of the reaction of EbPPK2 starting from **10a**. A) UV chromatogram at 254 nm. B) Extracted-ion chromatogram for **10a**-**10d**. C)-F) Mass spectrum for **10a**-**10d**.

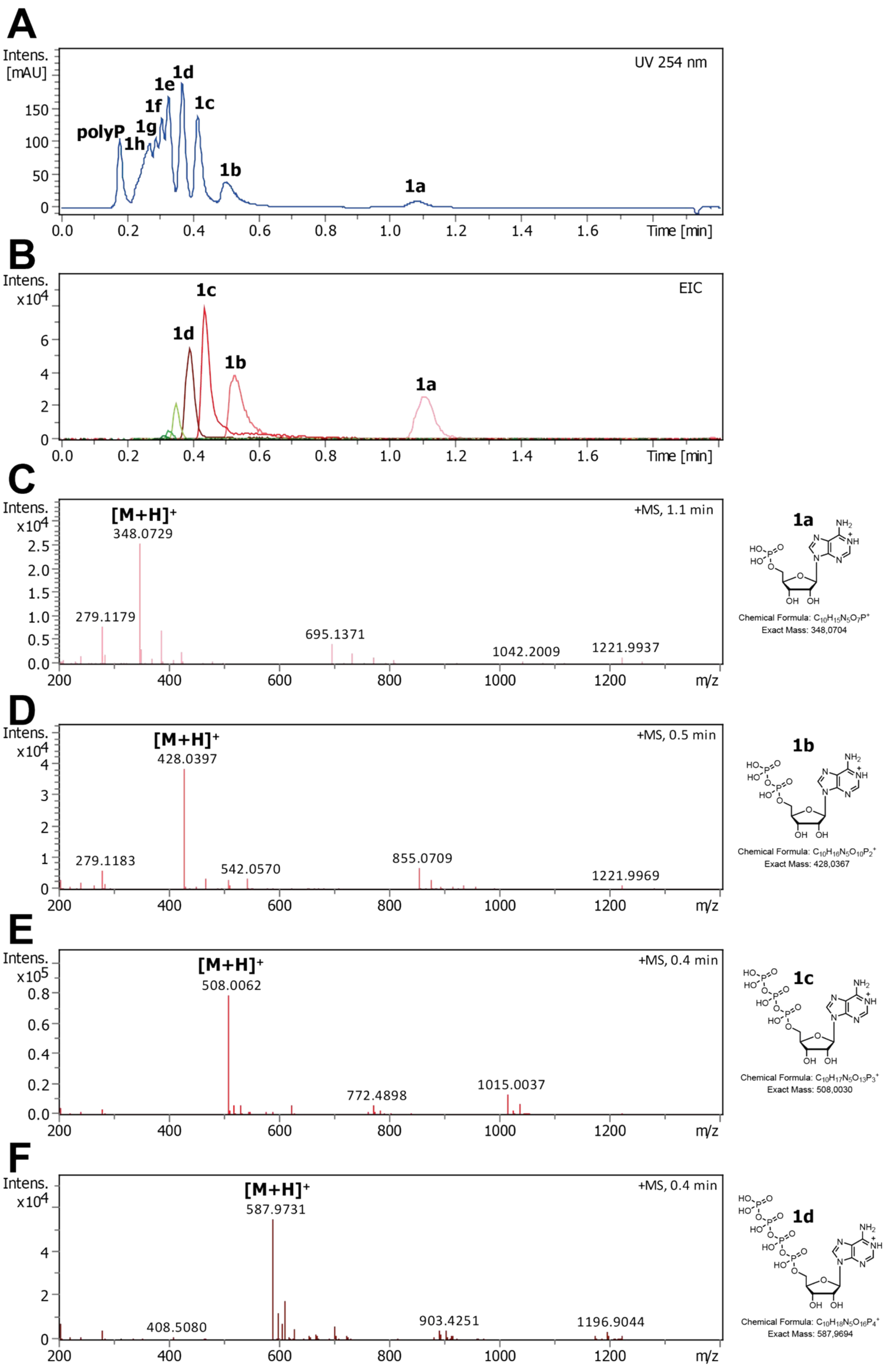


Fig. S19a: LC-TOF-MS analysis of the reaction of EbPPK2 starting from AMP (**1a**). With high enzyme load (43 µM) and long incubation times (20 h at 30 °C), the reaction can lead to ADP (**1b**), ATP (**1c**) and A4P (**1d**), A5P (**1e**), A6P (**1f**) A7P (**1g**) and A8P (**1h**). A) UV-chromatogram at 254 nm. B) Extracted-ion chromatogram for **1a**-**1d**. C)-F) Mass spectra for **1a**-**1d**. (Continued below)

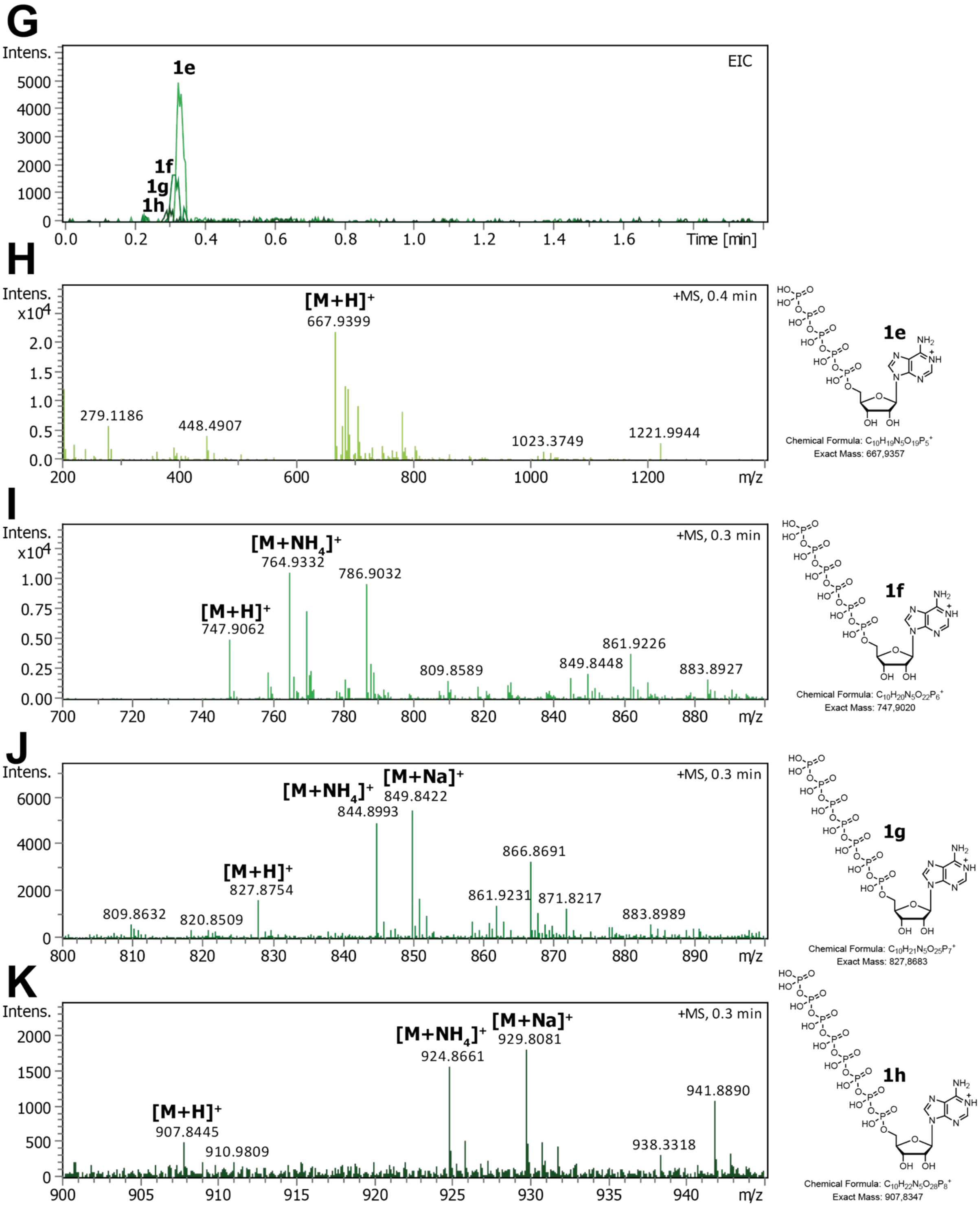


Fig. S19b: (continued) G) Extracted-ion chromatogram for **1e**-**1h**. H)-K) Mass spectra for **1e**-**1h**.

## Crystal structure of EbPPK2

### Table S2: Data collection and refinement statistics.

| | EbPPK2 with ACP and PolyP | EbPPK2 with PolyP |
|---|---|---|
| PDB code | **9IGQ** | **9IGR** |
| **Data collection** | | |
| Wavelength [Å] | 0.976 | 0.976 |
| Space group | P 21 21 21 | C 1 2 1 |
| Unit cell a, b,c [Å]<br>α. β, γ [°] | 77.89, 110.73 , 152.59,<br>90.00, 90.00, 90.00 | 160.47, 88.27, 125.34,<br>90.00, 101.15, 90.00 |
| Resolution range [Å] | 48.90 - 1.70 (1.79-1.70) | 45.11 - 2.31 (2.44-2.31) |
| Total reflections | 1,743,729 (290,953) | 495,604 (81,093) |
| Unique reflections | 143,136 (23,261) | 72,761 (11,484) |
| Multiplicity | 12.2 (12.5) | 6.81 (7.06) |
| Completeness (%) | 98.1 (99.8) | 96.6 (94.8) |
| I / sigma | 10.32 (1.45) | 0.996 (0.773) |
| R-meas (%) | 18.6 (209.6) | 0.999 (0.934) |
| R-pim | 5.33 (59.3) | 9.03 (0.98) |
| CC ½ | 0.998 (0.628) | 13.7 (1735.8) |
| CC* | 0.999 (0.878) | 5.25 (653.3) |
| Wilson B-factor | 18.53 | 50.64 |
| | | |
| | | |
| **Refinement** | | |
| Reflections in refinement | 144,529 | 72669 |
| Reflections in free set | 7,180 | 3729 |
| Rwork | 0.1670 | 0.1760 |
| Rfree | 0.1970 | 0.2130 |
| FSC average | 0.9728 | 0.9663 |
| RMSD bonds | 0.0143 | 0.0156 |
| RMSD angles | 2.2110 | 2.7470 |
| Ramachandran favoured (%) | 98.2 | 96.4 |
| Ramachandran allowed (%) | 1.8 | 3.6 |
| Ramachandran outliers (%) | 0.0 | 0.0 |
| Rotamer outliers | 1.0 | 3.7 |
| Clash score | 1.4 | 2.4 |
| MolProbity score | 0.87 | 1.7 |

Statistics for the last shell is given in parentheses (if unmerged data are available).

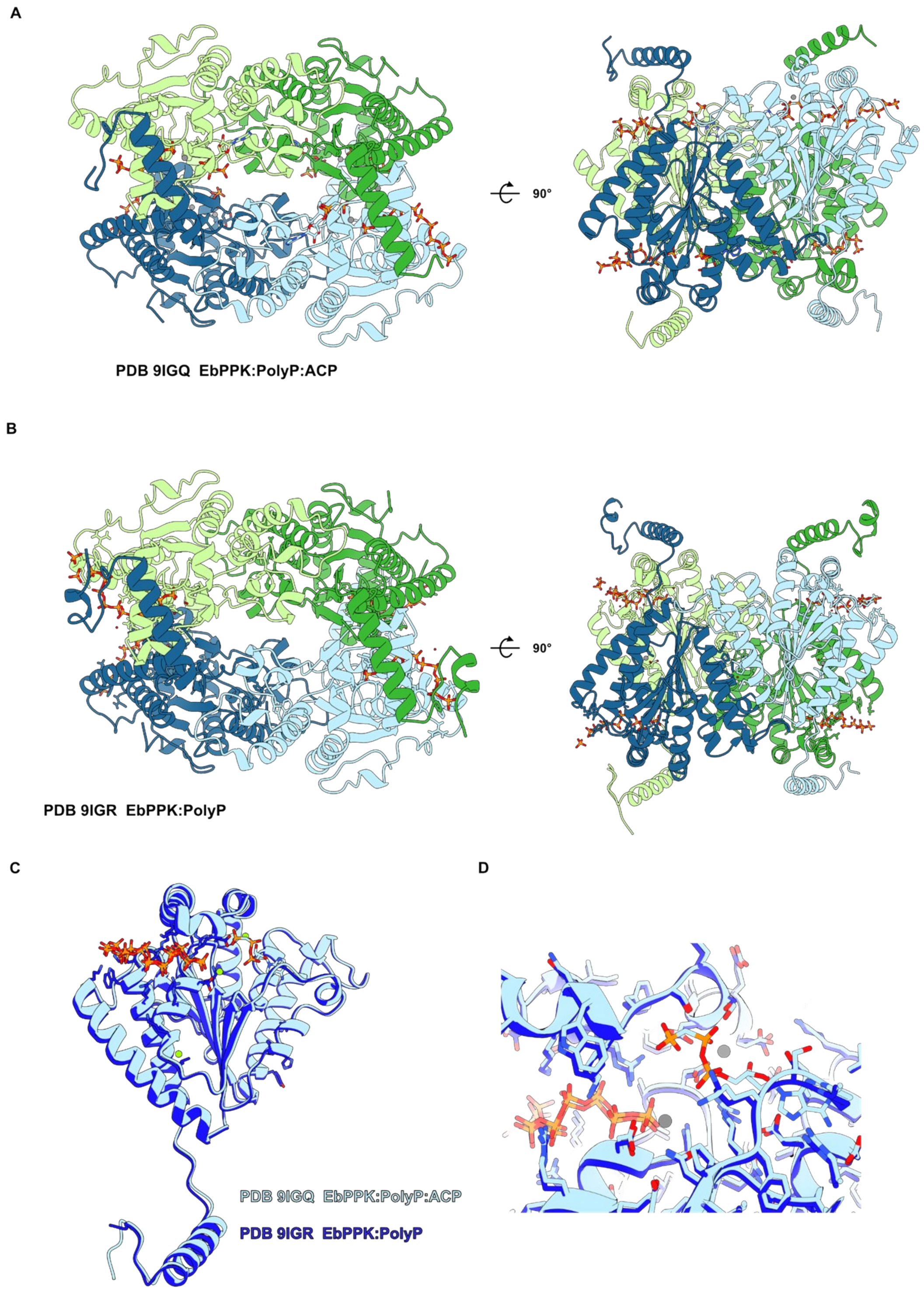


Fig. S20: Overview of the crystal structures of EbPPK2. A) Structure of EbPPK2 in complex with adenosine-5′-[(β,γ)-methyleno]triphosphate (ACP) and polyP (PDB: 9IGQ) at 1.7 Å resolution. B) Structure of EbPPK2 in complex with polyP (PDB: 9IGR) at 2.3 Å. C-D) Overlay of the two monomers of the EbPPK2 structures (PDB: 9IGQ and 9IGR) and overlay of the active site.

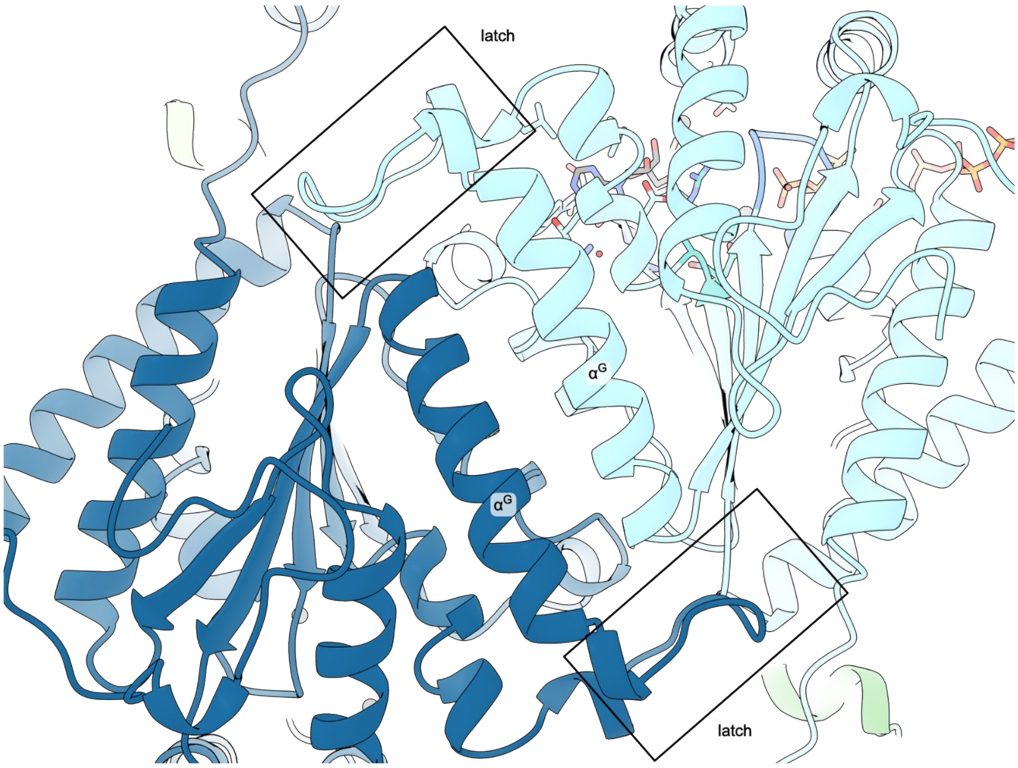


Fig. S21: Formation of the tetrameric structure from two homodimer protomers is aided by the latch loop.

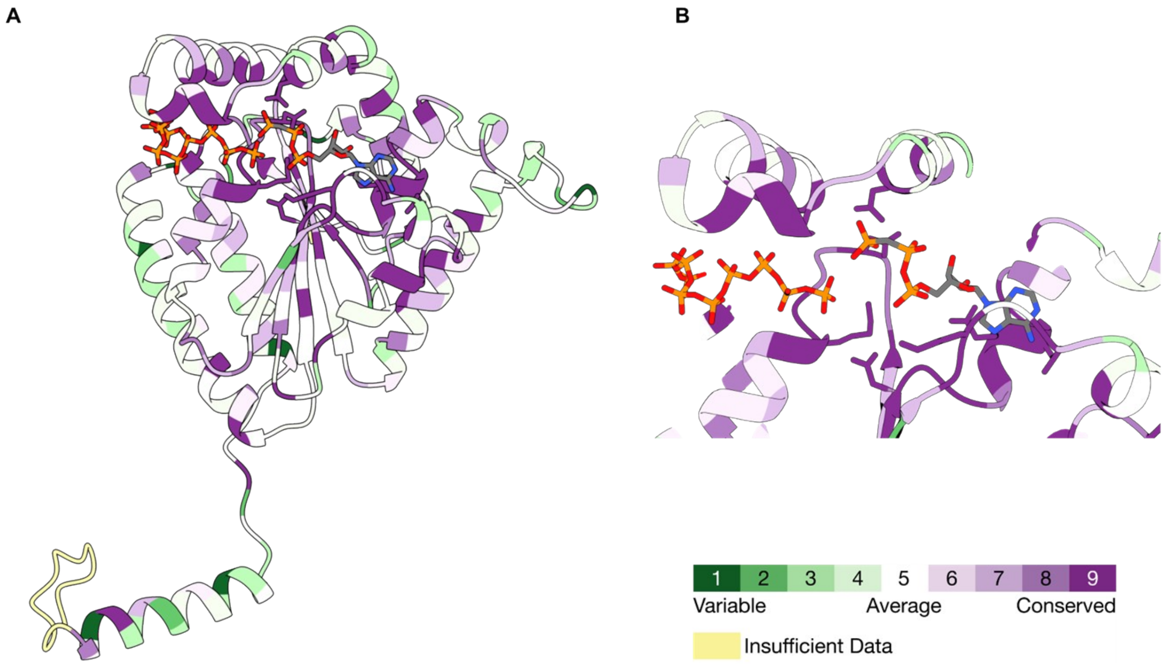


Fig. S22: Sequence conservation mapped onto the structure of EbPPK2 (PDB 9IGQ) using ConSurf.[16] A) Monomer of EbPPK2 (PDB: 9IGQ) and B) section showing the active site.

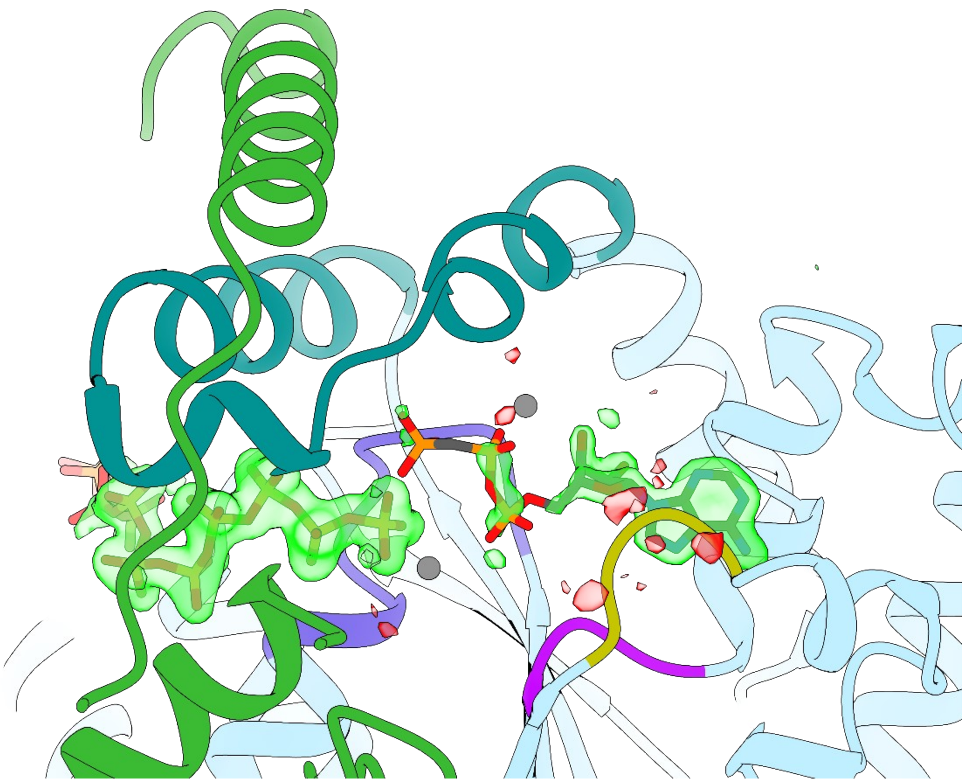

Fig. S23: Polder map of the ACP and polyphosphate ligand in EbPPK2 (9IGQ). The map was calculated omitting both ligands simultaneously and depicted at +/- 3 σ RMSD (green/red surface).

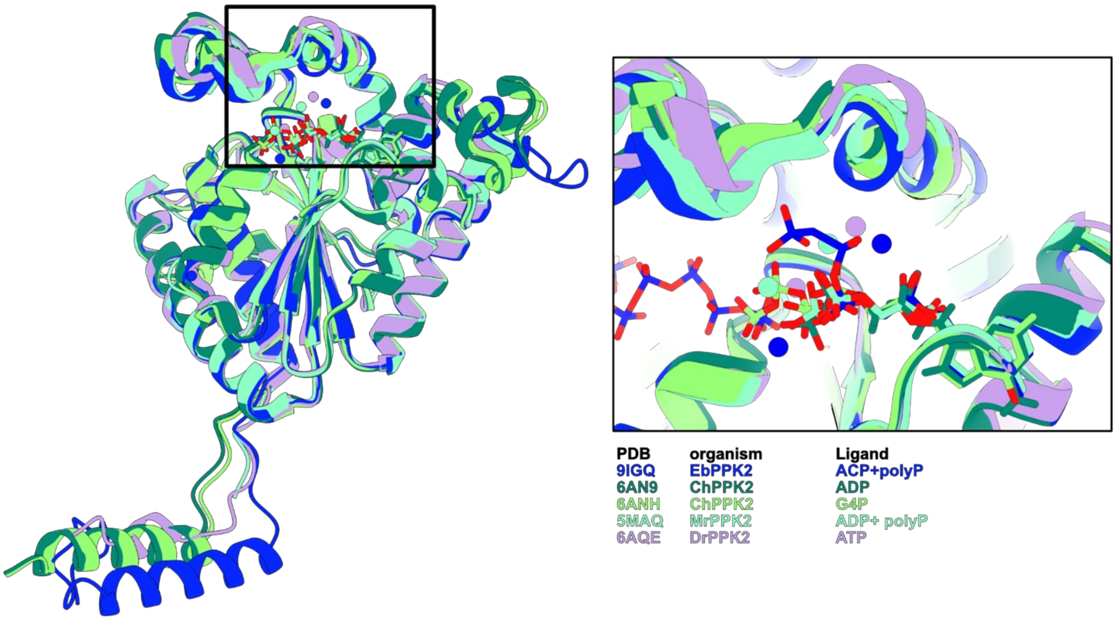


Fig. S24: Overlay of EbPPK2 (9IGQ) with related PPK2-III structures. The monomers show an overall comparable fold. The close-up of the active pocket shows that the lid module in EbPPK extends further into the pocket than is seen with the other enzymes. EbPPK2 in complex with polyP and ACP (this work) and the previously reported structures of *Cytophaga hutchinsonii* PPK2 (ChPPK2, 6AN9, 6ANH)[17], *Meiothermus*

*ruber* PPK2 (MrPPK, 5MAQ)[18] and *Deinococcus radiodurans* PPK2 (DrPPK2, 6AQE)[17].

```
CLUSTAL O(1.2.4) multiple sequence alignment


EbPPK2          ----------MINIYKIDKLNNFNLN-N-HKTDDYSLCKDKDTALELTQKNIQKIYDYQQ      48
DrPPK           ---------MDIDNYRVKPGKRVKLS-DWATNDDAGLSK--EEGQAQTAKLAGELAEWQE      48
MrPPK           -----------MKKYRVQPDGRFELK-RFDPDDTSAFEGGKQAALEALAVLNRRLEKLQE      48
ChPPK           MATDFSKLSKYVETLRVKPKQSIDLKKDFDTDYDHKMLTKEE-GEELLNLGISKLSEIQE      59
MmPPK           ----MKSKQLNTDLFKVQAKQQIRLK-NYDPASCNGFANRKE-AEHQLKQDIKELADLQY      54
                            .  ::.    . *.          :    : .         .: . *

                  latch-contact Walker A                  Nuc-loop   N7-contact
EbPPK2          KLYAEKKEGLIIAFQAMDAAGKDGTIREVLKALAPQGVHEKPFKSPSSTELAHDYLWRVH      108
DrPPK           RLYAEGKQSLLLILQARDAAGKDGAVKKVIGAFNPAGVQITSFKQPSAEELSHDFLWRIH      108
MrPPK           LLYAEGQHKVLVVLQAMDAGGKDGTIRVVFDGVNPSGVRVASFGVPTEQELARDYLWRVH      108
ChPPK           KLYASGTKSVLIVFQAMDAAGKDGTVKHIMTGLNPQGVKVTSFKVPSKIELSHDYLWRHY      119
MmPPK           QLYAENKRSVLIVFQAMDAAGKDGSIRHVFSGLNPQGCKVYSFKSPSANELDHDYLWRHY      114
                 ***.  . ::: :** **.****::: :: .. * * :   *  *:  ** :*:*** :

                         Walker B N6-contact    latch-loop
EbPPK2          NAVPEKGEITIFNRSHYEDVLIGKVKELYKFQNKADRID-----ENTVVDNRYEDIRNFE      163
DrPPK           QKAPAKGYVGVFNRSQYEDVLVTRVY-----------------DMIDDKTAKRRLEHIRHFE      153
MrPPK           QQVPRKGELVIFNRSHYEDVLVVRVK-----------------NLVPQQVWQKRYRHIREFE      153
ChPPK           VALPATGEIGIFNRSHYENVLVTRVHPEYLLSEQTSGVTAIEQVNQKFWDKRFQQINNFE      179
MmPPK           NKLPPRGEIGIFNRSHYENVLITRVHPEFLLNENLPDVHATNDITTEFWERRYRQIRDFE      174
                   *  * : :****:**:**: :*                        ..* ..*..**

                latch-contact                     lid
EbPPK2          KYLYNNSVRIIKIFLNVSKKEQAERFLSRIEEPEKNWKFSDSDFEERVYWDKYQQAFEDA      223
DrPPK           ELLTDNATRIVKVYLHISPEEQKERLQARLDNPGKHWKFNPGDLKDRSNWDKFNDVYEDA      213
MrPPK           RMLADEGTTILKFFLHISKDEQRQRLQERLDNPEKRWKFRMGDLEDRRLWDRYQEAYEAA      213
ChPPK           QHISENGTIVLKFFLHVSKKEQKKRFIERIELDTKNWKFSTGDLKERAHWKDYRNAYEDM      239
MmPPK           QTLTENGTTIIKFFLHLSKDEQKKRFMERIRKPAKNWKFSSADIRERQYWDDYQHAYEQA      234
                . : ::.. ::*.:*::* .** :*:  *:    *.***  .*:.:*  *. :...:*

                                                    clamp
EbPPK2          INATSTKDCPWYVVPADRKWYMRYVVSEIVVKTLEEMNPKYPTVTKETLERFEGYRTKLL      283
DrPPK           LT-TSTDDAPWYVVPADRKWYRDLVLSHILLGALKDMNPQFPAIDYDPSKVVIH------      266
MrPPK           IRETSTEYAPWYVIPANKNWYRNWLVSHILVETLEGLAMQYPQPETASEKIVIE------      267
ChPPK           LANTSTKQAPWFVIPADDKWFTRLLIAEIICTELEKLNLTFPTVSLEQKAELEKAKAELV      299
MmPPK           MRATSTEEAPWYIIPADNKWFTHVAIGNIIVETLQQMNIQMPEISSEEKDALKKAKKKLQ      294
                :  ***. .**:::**: :*:    :..*:   *: :    *         .

                clamp
EbPPK2          EEYNYDLDTIRPIEK      298
DrPPK           ---------------      266
MrPPK           ---------------      267
ChPPK           AEKSSD---------      305
MmPPK           NE-------------      296
```

Fig S25: Sequence alignment of EbPPK2 (UniProt: A0A3D5XRJ5), DrPPK (UniProt: Q9RY20), MrPPK (UniProt: M9XB82), ChPPK (UniProt: A0A6N4SMB5) and MmPPK (UniProt: A0A2T5C445) using Clustal Omega1.2.4.

**Expression and purification of EbPPK2 variants D127A and D127N**

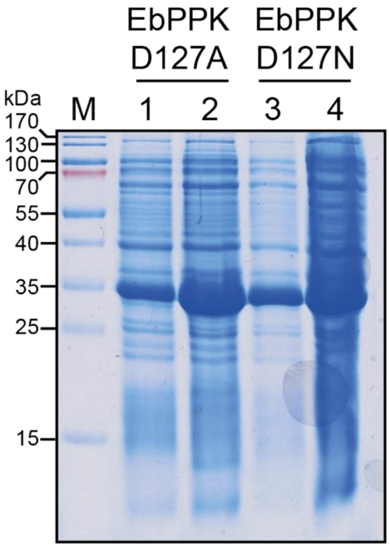


Fig S26: SDS-PAGE of EbPPK2 D127A and D127N induction. M: Marker, 1: EbPPK D127A before induction, 2: EbPPK D127A after induction, 3: EbPPK D127N before induction, 4: EbPPK D127N after induction.

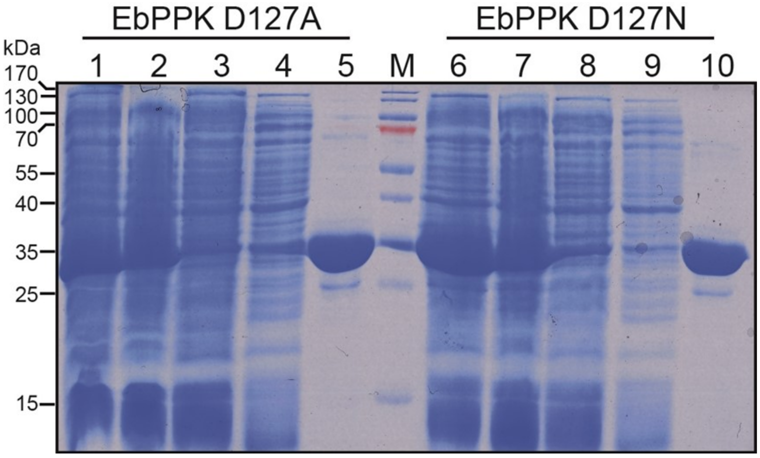


Figure S27: SDS-Page analysis of EbPPK2 D127A and D127N expression. M: Marker, EbPPK2 D127A: 1: Lysate, 2: Filtrate, 3: Flow-through, 4: Wash, 5: Eluate, EbPPK2 D127N: 6: Lysate, 7: Filtrate, 8: Flow-through, 9: Wash, 10: Eluate.

**Acquisition Parameter**

| | | | | | |
|---|---|---|---|---|---|
| Source Type | ESI | Ion Polarity | Positive | Set Nebulizer | 3.5 Bar |
| Focus | Active | Set Capillary | 4500 V | Set Dry Heater | 200 °C |
| Scan Begin | 300 m/z | Set End Plate Offset | -500 V | Set Dry Gas | 8.0 l/min |
| Scan End | 3000 m/z | Set Charging Voltage | 2000 V | Set Divert Valve | Waste |
| | | Set Corona | 0 nA | Set APCI Heater | 0 °C |

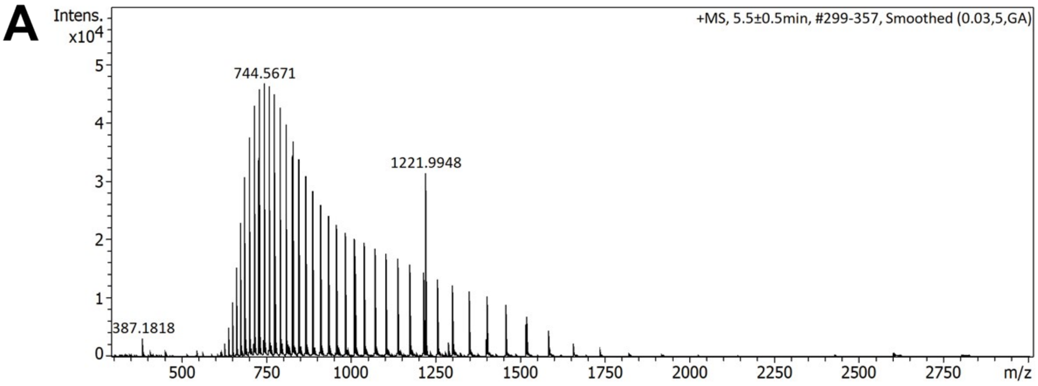


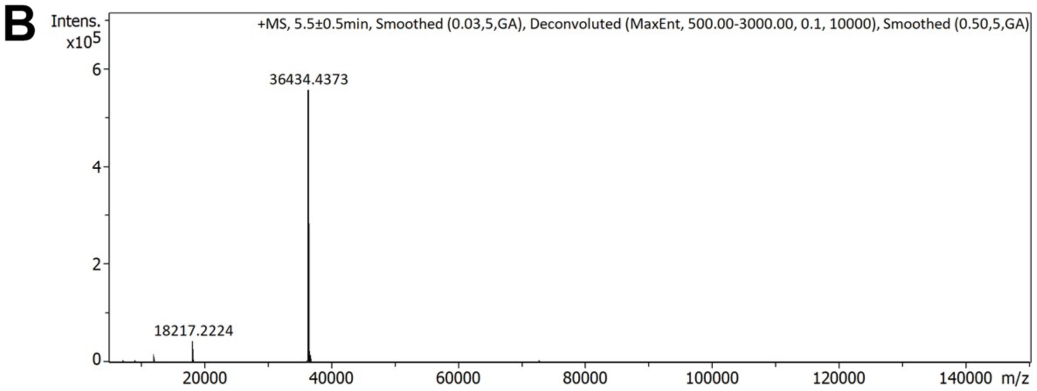


**m/z for A**

| # | m/z | Res. | S/N | I | I % | FWHM | Area |
|---|---|---|---|---|---|---|---|
| 1 | 675.7214 | 2809 | 437.4 | 22875 | 48.9 | 0.2405 | 5994 |
| 2 | 688.4507 | 2797 | 608.6 | 30749 | 65.7 | 0.2461 | 8194 |
| 3 | 701.6703 | 2763 | 737.5 | 37598 | 80.4 | 0.2539 | 10249 |
| 4 | 715.4085 | 2767 | 806.2 | 42967 | 91.9 | 0.2586 | 12091 |
| 5 | 729.6962 | 2756 | 781.6 | 45831 | 98.0 | 0.2648 | 13314 |
| 6 | 744.5671 | 2753 | 751.3 | 46766 | 100.0 | 0.2704 | 13426 |
| 7 | 760.0576 | 2754 | 748.4 | 46333 | 99.1 | 0.2760 | 13945 |
| 8 | 776.2080 | 2766 | 565.1 | 44960 | 96.1 | 0.2806 | 14654 |
| 9 | 793.0601 | 2764 | 527.0 | 42598 | 91.1 | 0.2870 | 12888 |
| 10 | 810.6607 | 2779 | 406.8 | 39733 | 85.0 | 0.2917 | 12566 |
| 11 | 829.0611 | 2782 | 342.8 | 36809 | 78.7 | 0.2981 | 11834 |
| 12 | 848.3189 | 2789 | 315.8 | 33768 | 72.2 | 0.3041 | 11350 |
| 13 | 868.4935 | 2793 | 287.8 | 30857 | 66.0 | 0.3110 | 10884 |
| 14 | 889.6523 | 2802 | 263.3 | 28330 | 60.6 | 0.3175 | 9732 |
| 15 | 911.8674 | 2797 | 242.0 | 25993 | 55.6 | 0.3260 | 9088 |
| 16 | 935.2226 | 2796 | 226.5 | 24073 | 51.5 | 0.3345 | 8505 |
| 17 | 959.8094 | 2805 | 213.0 | 22558 | 48.2 | 0.3422 | 8507 |
| 18 | 985.7229 | 2826 | 206.8 | 21332 | 45.6 | 0.3488 | 7823 |
| 19 | 1013.0756 | 2809 | 201.2 | 20274 | 43.4 | 0.3607 | 7686 |
| 20 | 1221.9948 | 16830 | 358.6 | 31457 | 67.3 | 0.0726 | 2764 |

**m/z for B (deconvoluted)**

| # | m/z | Res. | S/N | I | I % | FWHM | Area |
|---|---|---|---|---|---|---|---|
| 1 | 7287.0034 | 2385 | 7100.5 | 2521 | 0.5 | 3.0557 | 8633 |
| 2 | 9108.6867 | 2517 | 9346.5 | 3319 | 0.6 | 3.6188 | 14491 |
| 3 | 12144.8699 | 2624 | 32295.8 | 11467 | 2.1 | 4.6289 | 61774 |
| 4 | 18217.2224 | 2758 | 122519.0 | 43503 | 7.8 | 6.6050 | 307761 |
| 5 | 18236.0469 | 1921 | 5867.9 | 2084 | 0.4 | 9.4939 | 8545 |
| 6 | 18306.7434 | 2296 | 5944.2 | 2111 | 0.4 | 7.9749 | 15240 |
| 7 | 18345.7879 | 3336 | 3772.1 | 1339 | 0.2 | 5.5000 | 5827 |
| 8 | 36377.3806 | 3307 | 12706.2 | 4512 | 0.8 | 11.0003 | 41810 |
| 9 | 36390.2556 | 3908 | 22386.2 | 7949 | 1.4 | 9.3113 | 34727 |
| 10 | 36434.4373 | 2956 | 1570817.4 | 557753 | 100.0 | 12.3244 | 6461526 |
| 11 | 36471.0490 | 1989 | 107747.4 | 38258 | 6.9 | 18.3406 | 482813 |
| 12 | 36505.3567 | 2492 | 43082.7 | 15297 | 2.7 | 14.6510 | 139354 |
| 13 | 36534.1253 | 3290 | 45217.3 | 16055 | 2.9 | 11.1039 | 125407 |
| 14 | 36567.6861 | 3742 | 12261.2 | 4354 | 0.8 | 9.7721 | 23986 |
| 15 | 36571.9701 | 4156 | 12491.8 | 4435 | 0.8 | 8.8000 | 28236 |
| 16 | 36578.3941 | 5900 | 12076.7 | 4288 | 0.8 | 6.2000 | 19489 |
| 17 | 36613.3169 | 2723 | 64178.2 | 22788 | 4.1 | 13.4458 | 235544 |
| 18 | 36692.7307 | 2835 | 42380.6 | 15048 | 2.7 | 12.9424 | 179483 |
| 19 | 72869.4900 | 4563 | 8773.4 | 3115 | 0.6 | 15.9698 | 52984 |
| 20 | 73640.9279 | 4944 | 4543.2 | 1613 | 0.3 | 14.8957 | 25206 |

Figure S28: LC-TOF-MS analysis of WT-EbPPK2. A) Intact protein mass spectrum. B) Deconvoluted spectrum. Calculated average mass for WT-EbPPK2 36434.68 Da, found 36434.44 m/z. Note that the start methionine is removed.

**Acquisition Parameter**

| | | | | | |
|---|---|---|---|---|---|
| Source Type | ESI | Ion Polarity | Positive | Set Nebulizer | 3.5 Bar |
| Focus | Active | Set Capillary | 4500 V | Set Dry Heater | 200 °C |
| Scan Begin | 300 m/z | Set End Plate Offset | -500 V | Set Dry Gas | 8.0 l/min |
| Scan End | 3000 m/z | Set Charging Voltage | 2000 V | Set Divert Valve | Waste |
| | | Set Corona | 0 nA | Set APCI Heater | 0 °C |

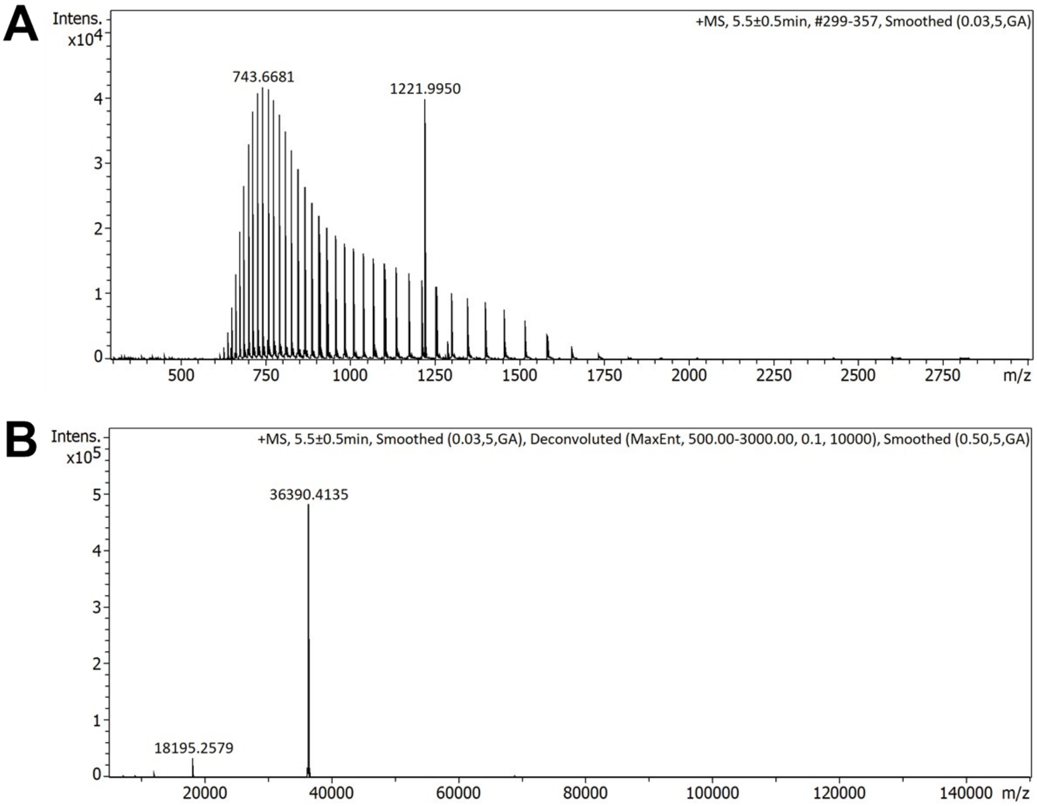


**m/z for A**

| # | m/z | Res. | S/N | I | I % | FWHM | Area |
|---|---|---|---|---|---|---|---|
| 1 | 674.9053 | 2798 | 289.7 | 19586 | 47.0 | 0.2412 | 5020 |
| 2 | 687.6202 | 2794 | 396.9 | 26483 | 63.5 | 0.2461 | 7254 |
| 3 | 700.8238 | 2783 | 509.6 | 32869 | 78.8 | 0.2518 | 9285 |
| 4 | 714.5456 | 2786 | 546.0 | 37858 | 90.8 | 0.2564 | 10367 |
| 5 | 728.8147 | 2776 | 579.5 | 40687 | 97.5 | 0.2626 | 11978 |
| 6 | 743.6681 | 2773 | 537.1 | 41715 | 100.0 | 0.2681 | 12642 |
| 7 | 759.1397 | 2770 | 523.7 | 41330 | 99.1 | 0.2741 | 12862 |
| 8 | 775.2702 | 2773 | 406.9 | 39761 | 95.3 | 0.2796 | 12680 |
| 9 | 792.1017 | 2780 | 388.2 | 37489 | 89.9 | 0.2849 | 11503 |
| 10 | 809.6819 | 2794 | 317.1 | 34798 | 83.4 | 0.2898 | 11428 |
| 11 | 828.0617 | 2801 | 267.6 | 32023 | 76.8 | 0.2957 | 10939 |
| 12 | 847.2949 | 2801 | 249.0 | 29078 | 69.7 | 0.3025 | 9839 |
| 13 | 867.4460 | 2800 | 225.0 | 26393 | 63.3 | 0.3098 | 8773 |
| 14 | 888.5763 | 2803 | 205.2 | 24019 | 57.6 | 0.3170 | 8531 |
| 15 | 910.7675 | 2816 | 188.7 | 21996 | 52.7 | 0.3234 | 8048 |
| 16 | 934.0935 | 2816 | 175.1 | 20220 | 48.5 | 0.3317 | 7150 |
| 17 | 958.6485 | 2827 | 164.3 | 18982 | 45.5 | 0.3391 | 6734 |
| 18 | 984.5298 | 2832 | 158.2 | 17785 | 42.6 | 0.3477 | 6973 |
| 19 | 1011.8519 | 2848 | 155.9 | 17043 | 40.9 | 0.3553 | 6508 |
| 20 | 1221.9950 | 16854 | 423.8 | 39858 | 95.6 | 0.0725 | 3459 |

**m/z for B (deconvoluted)**

| # | m/z | Res. | S/N | I | I % | FWHM | Area |
|---|---|---|---|---|---|---|---|
| 1 | 7278.1540 | 2442 | 4929.9 | 1762 | 0.4 | 2.9810 | 5981 |
| 2 | 9097.6516 | 2549 | 7108.4 | 2541 | 0.5 | 3.5688 | 11173 |
| 3 | 12130.1875 | 2689 | 23140.1 | 8273 | 1.7 | 4.5110 | 44913 |
| 4 | 18195.2579 | 2763 | 93035.5 | 33261 | 6.9 | 6.5852 | 231558 |
| 5 | 18214.6496 | 3036 | 5881.8 | 2103 | 0.4 | 6.0000 | 9021 |
| 6 | 18284.6900 | 2612 | 4086.2 | 1461 | 0.3 | 6.9997 | 8481 |
| 7 | 18323.7683 | 2955 | 5208.8 | 1862 | 0.4 | 6.2018 | 11539 |
| 8 | 36348.1834 | 363482 | 17118.8 | 6120 | 1.3 | 0.1000 | 18866 |
| 9 | 36390.4135 | 3007 | 1344910.6 | 480823 | 100.0 | 12.1027 | 5367894 |
| 10 | 36425.7271 | 8673 | 110762.5 | 39599 | 8.2 | 4.2000 | 122031 |
| 11 | 36459.3459 | 4740 | 45490.8 | 16264 | 3.4 | 7.6917 | 70049 |
| 12 | 36489.5880 | 3649 | 52889.0 | 18909 | 3.9 | 10.0000 | 135866 |
| 13 | 36504.4399 | 6294 | 22488.6 | 8040 | 1.7 | 5.8000 | 33611 |
| 14 | 36517.5409 | 6189 | 15685.1 | 5608 | 1.2 | 5.9000 | 24616 |
| 15 | 36522.0351 | 7722 | 15685.2 | 5608 | 1.2 | 4.7298 | 23772 |
| 16 | 36531.6866 | 5199 | 15578.7 | 5570 | 1.2 | 7.0267 | 22929 |
| 17 | 36568.4206 | 3585 | 37174.9 | 13291 | 2.8 | 10.2000 | 95061 |
| 18 | 36604.6899 | 4751 | 11455.1 | 4095 | 0.9 | 7.7042 | 21673 |
| 19 | 36648.3957 | 2973 | 45285.9 | 16190 | 3.4 | 12.3281 | 196353 |
| 20 | 68983.2559 | 6527 | 6884.1 | 2461 | 0.5 | 10.5687 | 27326 |

Figure S29: LC-TOF-MS analysis of EbPPK2 D127A. A) Intact protein mass spectrum. B) Deconvoluted spectrum. Calculated average mass for EbPPK2 D127A 36390.67 Da, found 36390.41 m/z. Note that the start methionine is removed.

**Acquisition Parameter**

| | | | | | |
|---|---|---|---|---|---|
| Source Type | ESI | Ion Polarity | Positive | Set Nebulizer | 3.5 Bar |
| Focus | Active | Set Capillary | 4500 V | Set Dry Heater | 200 °C |
| Scan Begin | 300 m/z | Set End Plate Offset | -500 V | Set Dry Gas | 8.0 l/min |
| Scan End | 3000 m/z | Set Charging Voltage | 2000 V | Set Divert Valve | Waste |
| | | Set Corona | 0 nA | Set APCI Heater | 0 °C |

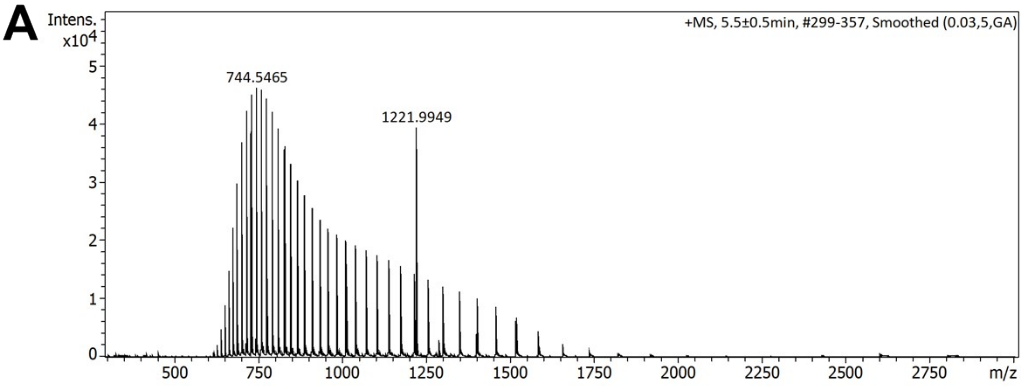


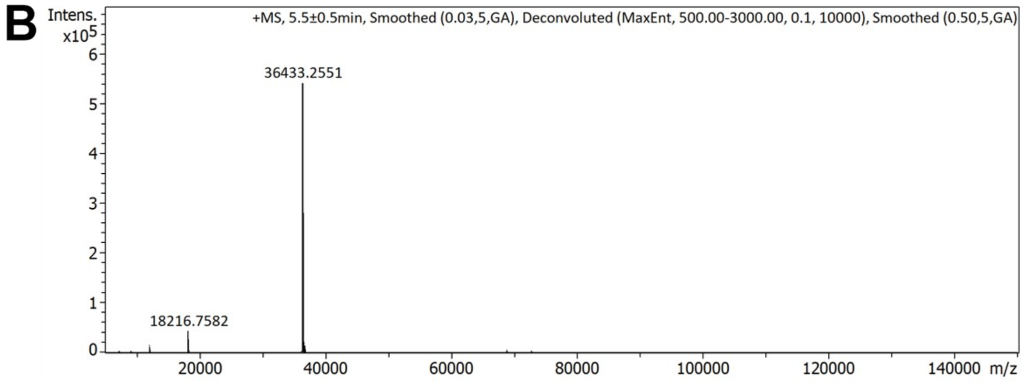


**m/z for A**

| # | m/z | Res. | S/N | I | I % | FWHM | Area |
|---|---|---|---|---|---|---|---|
| 1 | 675.7022 | 2818 | 347.9 | 22173 | 47.9 | 0.2398 | 5937 |
| 2 | 688.4312 | 2780 | 480.4 | 29760 | 64.3 | 0.2476 | 8195 |
| 3 | 701.6503 | 2781 | 566.6 | 36880 | 79.6 | 0.2523 | 10539 |
| 4 | 715.3878 | 2771 | 590.5 | 42201 | 91.1 | 0.2581 | 12192 |
| 5 | 729.6758 | 2771 | 618.8 | 45169 | 97.5 | 0.2633 | 13285 |
| 6 | 744.5465 | 2764 | 575.0 | 46319 | 100.0 | 0.2694 | 13949 |
| 7 | 760.0363 | 2776 | 554.9 | 45944 | 99.2 | 0.2738 | 14056 |
| 8 | 776.1847 | 2757 | 427.6 | 44432 | 95.9 | 0.2815 | 13926 |
| 9 | 793.0371 | 2781 | 400.6 | 42054 | 90.8 | 0.2851 | 13409 |
| 10 | 810.6375 | 2793 | 314.2 | 39133 | 84.5 | 0.2902 | 12805 |
| 11 | 829.0378 | 2793 | 266.9 | 36142 | 78.0 | 0.2968 | 12139 |
| 12 | 848.2953 | 2792 | 252.6 | 33124 | 71.5 | 0.3038 | 10368 |
| 13 | 868.4683 | 2813 | 232.0 | 30323 | 65.5 | 0.3087 | 10450 |
| 14 | 889.6249 | 2801 | 214.3 | 27743 | 59.9 | 0.3176 | 9837 |
| 15 | 911.8416 | 2829 | 200.5 | 25614 | 55.3 | 0.3224 | 9279 |
| 16 | 935.1971 | 2823 | 187.1 | 23647 | 51.1 | 0.3313 | 8843 |
| 17 | 959.7823 | 2801 | 175.8 | 22110 | 47.7 | 0.3426 | 8717 |
| 18 | 985.6943 | 2818 | 171.1 | 20996 | 45.3 | 0.3497 | 8172 |
| 19 | 1013.0450 | 2819 | 167.6 | 20058 | 43.3 | 0.3594 | 7954 |
| 20 | 1221.9949 | 16837 | 381.9 | 39450 | 85.2 | 0.0726 | 3473 |

**m/z for B (deconvoluted)**

| # | m/z | Res. | S/N | I | I % | FWHM | Area |
|---|---|---|---|---|---|---|---|
| 1 | 9108.4375 | 2541 | 8888.4 | 3253 | 0.6 | 3.5840 | 13848 |
| 2 | 12144.5406 | 2722 | 31113.8 | 11388 | 2.1 | 4.4611 | 57518 |
| 3 | 18216.7582 | 2832 | 118605.1 | 43410 | 8.0 | 6.4333 | 314538 |
| 4 | 18234.7425 | 2568 | 7801.7 | 2855 | 0.5 | 7.1000 | 14616 |
| 5 | 36375.6725 | 4910 | 12002.2 | 4393 | 0.8 | 7.4087 | 28294 |
| 6 | 36390.8669 | 4962 | 29219.4 | 10694 | 2.0 | 7.3338 | 38437 |
| 7 | 36433.2551 | 3044 | 1481742.3 | 542322 | 100.0 | 11.9676 | 4868781 |
| 8 | 36468.6938 | 3377 | 130679.0 | 47829 | 8.8 | 10.8000 | 357249 |
| 9 | 36498.5195 | 364985 | 53735.4 | 19667 | 3.6 | 0.1000 | 66599 |
| 10 | 36503.9959 | 365040 | 52055.4 | 19052 | 3.5 | 0.1000 | 15203 |
| 11 | 36532.0344 | 2671 | 62577.5 | 22904 | 4.2 | 13.6786 | 220915 |
| 12 | 36560.2564 | 3018 | 21217.4 | 7766 | 1.4 | 12.1129 | 40372 |
| 13 | 36564.7463 | 4744 | 21279.0 | 7788 | 1.4 | 7.7075 | 30777 |
| 14 | 36590.8659 | 4021 | 12546.2 | 4592 | 0.8 | 9.1000 | 30243 |
| 15 | 36597.5813 | 5462 | 12330.0 | 4513 | 0.8 | 6.7000 | 21443 |
| 16 | 36611.7935 | 3078 | 34343.2 | 12570 | 2.3 | 11.8951 | 106551 |
| 17 | 36648.5766 | 366486 | 14763.8 | 5404 | 1.0 | 0.1000 | 3767 |
| 18 | 36691.0001 | 4253 | 39191.6 | 14344 | 2.6 | 8.6281 | 106819 |
| 19 | 68982.4775 | 5280 | 14075.3 | 5152 | 0.9 | 13.0657 | 69628 |
| 20 | 72867.5349 | 4688 | 7860.2 | 2877 | 0.5 | 15.5449 | 49664 |

Figure S30: LC-TOF-MS analysis of EbPPK2 D127N. A) Intact protein mass spectrum. B) Deconvoluted spectrum. Calculated average mass for EbPPK2 D127N 36433.70 Da, found 36433.26 m/z. Note that the start methionine is removed.

## HPLC analysis of EbPPK2 variant-catalysed reactions

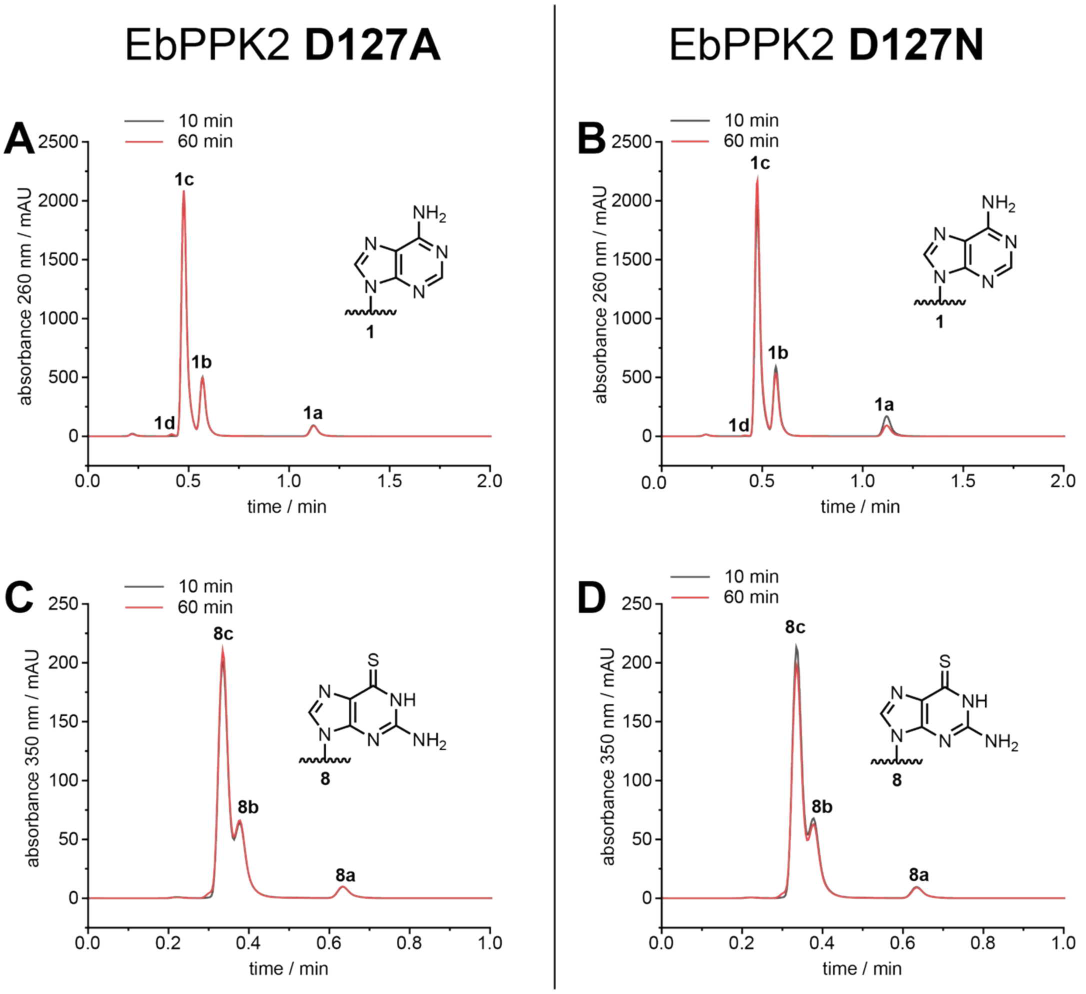


Fig. S31: A)-D) Representative HPLC analysis (method B) of the reaction of EbPPK2 variants (D127A or D127N) starting from indicated NMP (**1a** or **8a**).

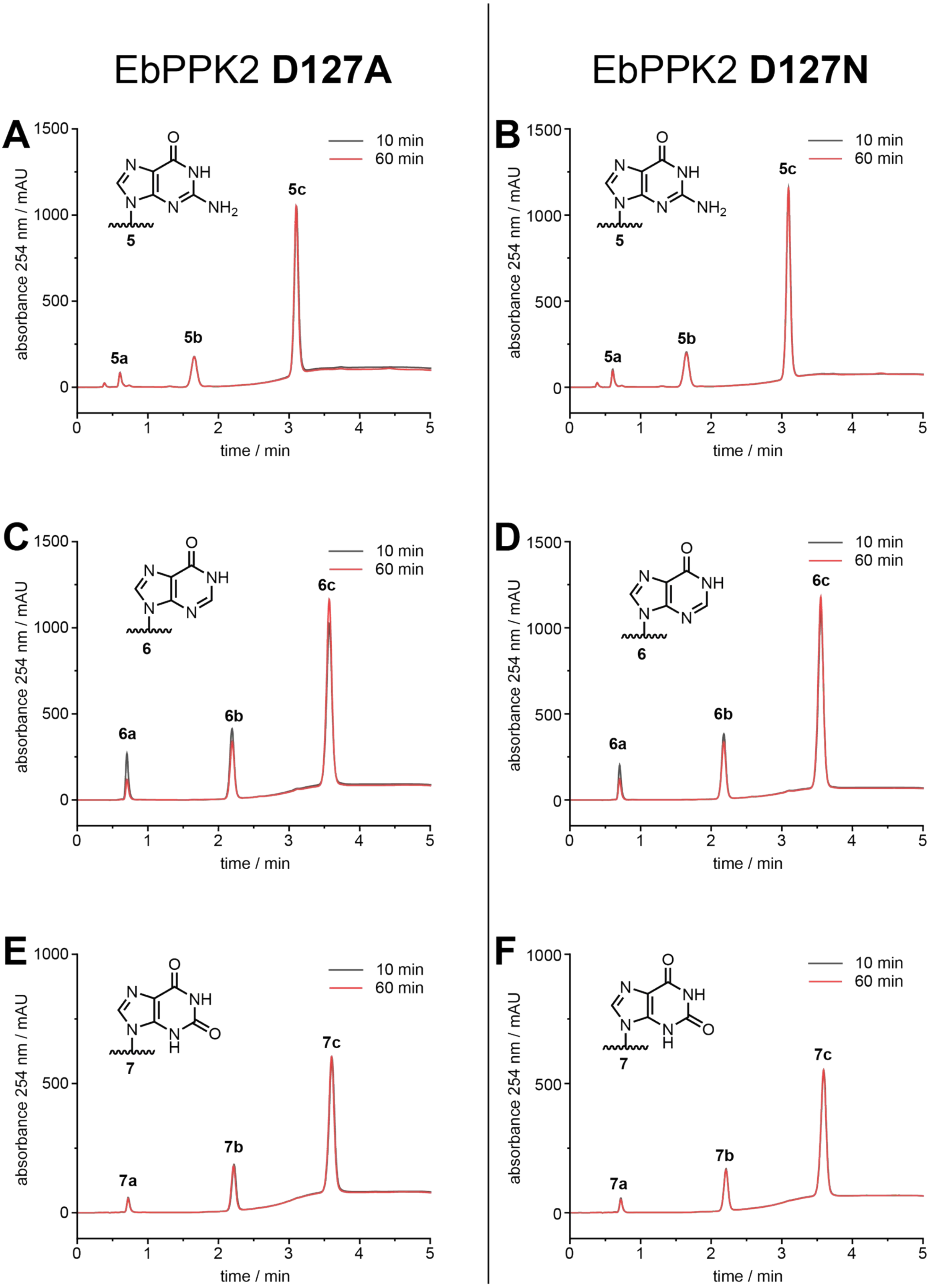


Fig. S32: A)-F) Representative HPLC analysis (method A) of the reaction of EbPPK2 starting from indicated AMP analogues (**5a-7a**).

**Table S3: Conversions of EbPPK2 variants D127A or D127N.** Reactions starting from **1a**, **5a-8a**. Average conversion and standard deviation of three independent experiments. Related to figure 5.

| **EbPPK D127A** | **d** (N4P) | | **c** (NTP) | | **b** (NDP) | | **a** (NMP) | |
|---|---|---|---|---|---|---|---|---|
| **10 min** | conv. (%) | ± SD | conv. (%) | ± SD | conv. (%) | ± SD | conv. (%) | ± SD |
| | | | | | | | | |
| **1** | | | 72,3 | 2,06 | 21,7 | 0,89 | 5,9 | |
| **5** | | | 76,7 | 0,42 | 18,5 | 0,10 | 4,8 | |
| **6** | | | 67,7 | 0,70 | 22,6 | 0,63 | 9,7 | |
| **7** | | | 75,7 | 0,24 | 19,7 | 0,41 | 4,6 | |
| **8** | | | 70,3 | 0,94 | 25,2 | 0,43 | 4,6 | |

| **EbPPK D127A** | **d** (N4P) | | **c** (NTP) | | **b** (NDP) | | **a** (NMP) | |
|---|---|---|---|---|---|---|---|---|
| **60 min** | conv. (%) | ± SD | conv. (%) | ± SD | conv. (%) | ± SD | conv. (%) | ± SD |
| | | | | | | | | |
| **1** | 0,47 | 0,04 | 74,4 | 0,31 | 20,4 | 0,23 | 4,7 | 0,07 |
| **5** | | | 76,6 | 0,32 | 18,8 | 0,14 | 4,6 | 0,30 |
| **6** | | | 76,9 | 0,32 | 18,6 | 0,05 | 4,5 | 0,35 |
| **7** | | | 76,1 | 0,35 | 19,6 | 0,12 | 4,3 | 0,29 |
| **8** | | | 70,4 | 0,53 | 25,1 | 0,35 | 4,5 | 0,36 |

| **EbPPK D127N** | **d** (N4P) | | **c** (NTP) | | **b** (NDP) | | **a** (NMP) | |
|---|---|---|---|---|---|---|---|---|
| **10 min** | conv. (%) | ± SD | conv. (%) | ± SD | conv. (%) | ± SD | conv. (%) | ± SD |
| | | | | | | | | |
| **1** | | | 68,2 | 0,29 | 22,8 | 0,10 | 9,0 | |
| **5** | | | 76,7 | 0,32 | 18,7 | 0,12 | 4,6 | |
| **6** | | | 72,9 | 1,21 | 20,7 | 0,49 | 6,4 | |
| **7** | | | 75,6 | 0,16 | 20,0 | 0,17 | 4,4 | |
| **8** | | | 70,1 | 0,19 | 25,6 | 0,35 | 4,3 | |

| **EbPPK D127N** | **d** (N4P) | | **c** (NTP) | | **b** (NDP) | | **a** (NMP) | |
|---|---|---|---|---|---|---|---|---|
| **60 min** | conv. (%) | ± SD | conv. (%) | ± SD | conv. (%) | ± SD | conv. (%) | ± SD |
| | | | | | | | | |
| **1** | 0,09 | 0,16 | 74,5 | 0,26 | 20,6 | 0,43 | 4,8 | 0,08 |
| **5** | | | 77,0 | 0,10 | 18,7 | 0,21 | 4,3 | 0,19 |
| **6** | | | 76,3 | 1,09 | 18,5 | 0,05 | 5,1 | 1,07 |
| **7** | | | 76,1 | 0,12 | 19,7 | 0,12 | 4,2 | 0,19 |
| **8** | | | 70,7 | 0,29 | 25,1 | 0,56 | 4,1 | 0,73 |
| | 0,09 | 0,16 | 74,5 | 0,26 | 20,6 | 0,43 | 4,8 | 0,08 |

## References


1. Mitton-Fry, R. M.; Eschenbach, J.; Schepers, H.; Rasche, R.; Erguven, M.; Kummel, D.; Rentmeister, A.; Cornelissen, N. V., Chemo-enzymatic production of base-modified ATP analogues for polyadenylation of RNA. *Chem. Sci.* **2024,** *15* (32), 13068-13073.
2. Michailidou, F.; Klöcker, N.; Cornelissen, N. V.; Singh, R. K.; Peters, A.; Ovcharenko, A.; Kummel, D.; Rentmeister, A., Engineered SAM Synthetases for Enzymatic Generation of AdoMet Analogs with Photocaging Groups and Reversible DNA Modification in Cascade Reactions. *Angew. Chem. Int. Ed.* **2021,** *60* (1), 480-485.
3. Cianci, M.; Bourenkov, G.; Pompidor, G.; Karpics, I.; Kallio, J.; Bento, I.; Roessle, M.; Cipriani, F.; Fiedler, S.; Schneider, T. R., P13, the EMBL macromolecular crystallography beamline at the low-emittance PETRA III ring for high- and low-energy phasing with variable beam focusing. *J. Synchrotron Radiat.* **2017,** *24*, 323-332.
4. Kabsch, W., Xds. *Acta Crystallogr. D Biol. Crystallogr.* **2010,** *66,* 125-32.
5. Brehm, W.; Trivino, J.; Krahn, J. M.; Uson, I.; Diederichs, K., XDSGUI: a graphical user interface for XDS, SHELX and ARCIMBOLDO. *J. Appl. Crystallogr.* **2023,** *56*, 1585-1594.
6. Agirre, J.; Atanasova, M.; Bagdonas, H.; Ballard, C. B.; Basle, A.; Beilsten-Edmands, J.; Borges, R. J.; Brown, D. G.; Burgos-Marmol, J. J.; Berrisford, J. M.; Bond, P. S.; Caballero, I.; Catapano, L.; Chojnowski, G.; Cook, A. G.; Cowtan, K. D.; Croll, T. I.; Debreczeni, J. E.; Devenish, N. E.; Dodson, E. J.; Drevon, T. R.; Emsley, P.; Evans, G.; Evans, P. R.; Fando, M.; Foadi, J.; Fuentes-Montero, L.; Garman, E. F.; Gerstel, M.; Gildea, R. J.; Hatti, K.; Hekkelman, M. L.; Heuser, P.; Hoh, S. W.; Hough, M. A.; Jenkins, H. T.; Jimenez, E.; Joosten, R. P.; Keegan, R. M.; Keep, N.; Krissinel, E. B.; Kolenko, P.; Kovalevskiy, O.; Lamzin, V. S.; Lawson, D. M.; Lebedev, A. A.; Leslie, A. G. W.; Lohkamp, B.; Long, F.; Maly, M.; McCoy, A. J.; McNicholas, S. J.; Medina, A.; Millan, C.; Murray, J. W.; Murshudov, G. N.; Nicholls, R. A.; Noble, M. E. M.; Oeffner, R.; Pannu, N. S.; Parkhurst, J. M.; Pearce, N.; Pereira, J.; Perrakis, A.; Powell, H. R.; Read, R. J.; Rigden, D. J.; Rochira, W.; Sammito, M.; Sanchez Rodriguez, F.; Sheldrick, G. M.; Shelley, K. L.; Simkovic, F.; Simpkin, A. J.; Skubak, P.; Sobolev, E.; Steiner, R. A.; Stevenson, K.; Tews, I.; Thomas, J. M. H.; Thorn, A.; Valls, J. T.; Uski, V.; Uson, I.; Vagin, A.; Velankar, S.; Vollmar, M.; Walden, H.; Waterman, D.; Wilson, K. S.; Winn, M. D.; Winter, G.; Wojdyr, M.; Yamashita, K., The CCP4 suite: integrative software for macromolecular crystallography. *Acta Crystallogr. D Struct. Biol.* **2023,** *79*, 449-461.
7. Krissinel, E.; Uski, V.; Lebedev, A.; Winn, M.; Ballard, C., Distributed computing for macromolecular crystallography. *Acta Crystallogr. D Struct. Biol.* **2018,** *74*, 143-151.
8. Jumper, J.; Evans, R.; Pritzel, A.; Green, T.; Figurnov, M.; Ronneberger, O.; Tunyasuvunakool, K.; Bates, R.; Zidek, A.; Potapenko, A.; Bridgland, A.; Meyer, C.; Kohl, S. A. A.; Ballard, A. J.; Cowie, A.; Romera-Paredes, B.; Nikolov, S.; Jain, R.; Adler, J.; Back, T.; Petersen, S.; Reiman, D.; Clancy, E.; Zielinski, M.; Steinegger, M.; Pacholska, M.; Berghammer, T.; Bodenstein, S.; Silver, D.; Vinyals, O.; Senior, A. W.; Kavukcuoglu, K.; Kohli, P.; Hassabis, D., Highly accurate protein structure prediction with AlphaFold. *Nature* **2021,** *596* (7873), 583-589.
9. McCoy, A. J.; Grosse-Kunstleve, R. W.; Adams, P. D.; Winn, M. D.; Storoni, L. C.; Read, R. J., Phaser crystallographic software. *J. Appl. Crystallogr.* **2007,** *40*, 658-674.
10. Murshudov, G. N.; Skubak, P.; Lebedev, A. A.; Pannu, N. S.; Steiner, R. A.; Nicholls, R. A.; Winn, M. D.; Long, F.; Vagin, A. A., REFMAC5 for the refinement of macromolecular crystal structures. *Acta Crystallogr. D Biol. Crystallogr.* **2011,** *67*, 355-67.
11. Long, F.; Nicholls, R. A.; Emsley, P.; Graaeulis, S.; Merkys, A.; Vaitkus, A.; Murshudov, G. N., AceDRG: a stereochemical description generator for ligands. *Acta Crystallogr. D Struct. Biol.* **2017,** *73*, 112-122.
12. Meng, E. C.; Goddard, T. D.; Pettersen, E. F.; Couch, G. S.; Pearson, Z. J.; Morris, J. H.; Ferrin, T. E., UCSF ChimeraX: Tools for structure building and analysis. *Protein Sci.* **2023,** *32* (11), e4792.
13. Liebschner, D.; Afonine, P. V.; Moriarty, N. W.; Poon, B. K.; Sobolev, O. V.; Terwilliger, T. C.; Adams, P. D., Polder maps: improving OMIT maps by excluding bulk solvent. *Acta Crystallogr. D: Struct. Biol.* **2017,** *73*, 148-157.
14. Liebschner, D.; Afonine, P. V.; Baker, M. L.; Bunkoczi, G.; Chen, V. B.; Croll, T. I.; Hintze, B.; Hung, L. W.; Jain, S.; McCoy, A. J.; Moriarty, N. W.; Oeffner, R. D.; Poon, B. K.; Prisant, M. G.; Read, R. J.; Richardson, J. S.; Richardson, D. C.; Sammito, M. D.; Sobolev, O. V.; Stockwell, D. H.; Terwilliger, T. C.; Urzhumtsev, A. G.; Videau, L. L.; Williams, C. J.; Adams, P. D., Macromolecular structure determination using X-rays, neutrons and electrons: recent developments in Phenix. *Acta Crystallogr. D: Struct. Biol.* **2019,** *75,* 861-877.
15. Mautjana, N. A.; Looi, D. W.; Eyler, J. R.; Brajter-Toth, A., Sensitivity of positive ion mode electrospray ionization mass spectrometry (ESI MS) in the analysis of purine bases in ESI MS and on-line electrochemistry ESI MS (EC/ESI MS). *Electrochimica Acta* **2009,** *55* (1), 52-58.
16. Ben Chorin, A.; Masrati, G.; Kessel, A.; Narunsky, A.; Sprinzak, J.; Lahav, S.; Ashkenazy, H.; Ben-Tal, N., ConSurf-DB: An accessible repository for the evolutionary conservation patterns of the majority of PDB proteins. *Protein Sci.* **2020,** *29* (1), 258-267.
17. Nocek, B. P.; Khusnutdinova, A. N.; Ruszkowski, M.; Flick, R.; Burda, M.; Batyrova, K.; Brown, G.; Mucha, A.; Joachimiak, A.; Berlicki, Ł.; Yakunin, A. F., Structural Insights into Substrate Selectivity and Activity of Bacterial Polyphosphate Kinases. *ACS Catal.* **2018,** *8* (11), 10746-10760.
18. Parnell, A. E.; Mordhorst, S.; Kemper, F.; Giurrandino, M.; Prince, J. P.; Schwarzer, N. J.; Hofer, A.; Wohlwend, D.; Jessen, H. J.; Gerhardt, S.; Einsle, O.; Oyston, P. C. F.; Andexer, J. N.; Roach, P. L., Substrate recognition and mechanism revealed by ligand-bound polyphosphate kinase 2 structures. *Proc. Natl. Acad. Sci. U.S.A.* **2018,** *115* (13), 3350-3355.
